# The Diarylprolinol Silyl Ethers: After 20 Years Still Opening New Doors in Asymmetric Catalysis

**DOI:** 10.1002/anie.202526146

**Published:** 2026-02-12

**Authors:** Enrico Marcantonio, René Slot Bitsch, Karl Anker Jørgensen

**Affiliations:** ^1^ Department of Chemistry Aarhus University Aarhus Denmark

**Keywords:** diarylprolinol silyl ethers, dual catalysis, electrochemistry, higher‐order cycloadditions, organocatalysis, photochemistry, total synthesis

## Abstract

*A new chapter starts now*. Since its discovery in 2005, the diarylprolinol silyl ether catalytic concept has emerged as a general and reliable aminocatalytic tool for the synthesis of enantioenriched molecules. Recently its combination with emerging technologies, as well as its application in more complex molecular systems has opened new avenues for novel enantioenriched scaffolds. In this review, we will highlight these recent developments, unfolding five primary categories that define new horizons in the use of diarylprolinol silyl ethers: Photochemical‐, electrochemical‐, dual‐catalytic transformations, higher‐order cycloadditions and applications in total synthesis of complex natural products.

## Graphical abstract

**Catalysis Rules!** The year 2025 marks the 20^th^ anniversary of diarylprolinol silyl ethers in asymmetric organocatalysis. During the first decade after their discovery, these catalysts have been established as one of the most versatile tools in aminocatalysis. Although now considered mature, recent years have witnessed renewed innovation. We outlined these developments, demonstrating that this remains a rapidly evolving field.

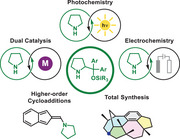

## 1 Introduction

In nature, many systems reach full maturity only after 20 years—from oak trees that begin to bear fruits, to coral reefs that stabilize into complex ecosystems. Humans themselves typically complete their physical maturation around the age of 20, reaching a phase of full capability and resilience.

Twenty years after their discovery, the diarylprolinol silyl ether organocatalysts have reached a level of maturity that does not mark an endpoint, but rather a new chapter from which new transformations continue to emerge. This review highlights the recent directions in asymmetric catalysis in which the diarylprolinol silyl ethers have played a pivotal role, further expanding the landscape of aminocatalysis.

**FIGURE 1:**
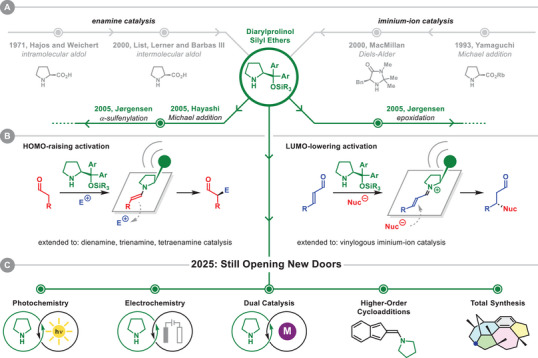
(A) Historical lineup of enamine and iminium‐ion catalysis. (B) Classical activation modes using diarylprolinol silyl ethers in enamine and iminium‐ion catalysis. (C) New horizons in diarylprolinol silyl ether catalysis.

Organocatalysis represents one of the major pillars of asymmetric catalysis along with enzymatic‐ and transition‐metal catalysis [1, 2]. The success of this field arises largely from the ability to design new enantioselective transformations in a conceptually straightforward manner, through the application of generic modes of activation [3, 4]. The catalytic generation of enamines and iminium ions, as transient covalent intermediates, through the condensation of chiral amines with carbonyl compounds, is defined as aminocatalysis [5]. From an historical perspective, the first examples involving enamine catalysis were independently reported by Weichert and Hajos in 1971 [6, 7], while Yamaguchi demonstrated the first organocatalytic iminium‐ion‐mediated transformation in 1993 (Figure 1A) [8]. The field gained momentum at the turn of the millennium, when the groups of List, Lerner, and Barbas III, and MacMillan independently introduced breakthrough aminocatalytic asymmetric transformations—an enamine‐mediated aldol reaction and an iminium‐ion‐mediated Diels–Alder reaction, respectively [9, 10].

Among the numerous chiral amines introduced since then, the diarylprolinol silyl ethers, disclosed in 2005, rapidly distinguished themselves as exceptionally reliable and broadly applicable chiral organocatalysts. That year, our research group and the Hayashi group independently reported the use of diarylprolinol silyl ethers as catalysts for the activation of aldehydes via chiral enamine intermediates, specifically in the α‐sulfenylation of aldehydes and the Michael addition of aldehydes to nitroalkenes, respectively [11, 12]. A few months later, the first application of the catalyst in iminium‐ion catalysis was reported by our group in the stereoselective epoxidation of α,β‐unsaturated aldehydes [13]. Since then, the research community has developed a myriad of aminocatalyzed transformations, establishing covalent activation of aldehydes as a reliable tool for generating new bonds and stereocenters at both the α‐ and β‐positions [14, 15].

In enamine catalysis, the chiral aminocatalyst condenses with an aldehyde giving rise to an enamine intermediate, resulting in an increase of the HOMO energy compared to the substrate enol‐tautomer and, thereby, higher nucleophilicity (HOMO‐raising activation). The enamine can now react in an enantioselective fashion with different electrophiles affording α‐functionalized aldehydes (Figure 1B, left). In contrast, in iminium‐ion catalysis, the condensation of the aminocatalyst with an α,β‐unsaturated aldehyde gives rise to an iminium‐ion intermediate featuring a reduced LUMO energy and improved electrophilicity, if compared to the starting α,β‐unsaturated aldehyde (LUMO‐lowering activation). The iminium‐ion intermediate allows for the enantioselective β‐functionalization with suitable nucleophilic species (Figure 1B, right).

The high enantioselectivity observed in aminocatalytic transformations is attributable to the well‐defined geometry of the covalently bound catalyst–substrate intermediates. Both in the case of enamine and iminium‐ion intermediates, the exocyclic group of the pyrrolidine moiety provides the steric bias responsible for the reliable enantioselectivity, due to the predictable facial discrimination of the transient intermediate in aminocatalytic transformations [16, 17]. These activation concepts extend to more complex intermediates, such as dienamines [18, 19], trienamines [20, 21], and tetraenamines [22], as well as vinylogous and bis‐vinylogous iminium ions [23, 24, 25], now considered classic modes of organocatalytic activation.

During the first decade after its discovery, diarylprolinol silyl ether has established itself as one of the most reliable catalytic tools in aminocatalysis, allowing for novel and unprecedented reactions in asymmetric catalysis—an impact reflected in the extensive body of articles, reviews, and perspectives devoted to it [26, 27, 28, 29, 30, 31]. Although now considered fully mature, recent years have witnessed renewed innovation. The classical activation modes have been integrated with emerging technologies such as photo‐ and electrochemistry. Their synergistic use with other systems, and application to increasingly complex substrates, such as higher‐order cycloadditions have further expanded their synthetic utility. These developments opened novel reaction concepts generating previously unachievable bond‐connections and demonstrate that this is a rapidly evolving field.

This review does not aim to provide a comprehensive overview of all existing transformations involving diarylprolinol silyl ethers. Instead, it offers a glance at the most innovative and recent developments enabled by the strategic use of this catalytic platform (Figure 1C). We have identified five primary categories that define new horizons in the use of diarylprolinol silyl ethers: Photochemical‐ and electrochemical transformations, dual catalytic systems, higher‐order cycloadditions, and application in total synthesis of complex natural products. Beyond these categories, some selected additional examples involving classical aminocatalytic activation modes will also be presented.

## 2 Photochemical Transformations

Catalytic enantioselective radical reactions have experienced tremendous growth over the past decade [32], largely driven by the continuous advances in light‐mediated strategies that enable radical generation under mild reaction conditions [33]. Among these, photoredox catalysis has emerged as the most widely used approach for producing radicals in a controlled and efficient manner [34, 35, 36].

The pioneering work of most recent photoredox catalysis can be attributed to MacMillan, who in 2008 applied the concept of photoexcitation of ruthenium‐based polypyridyl catalyst to promote an organocatalyzed, and enantioselective, radical α‐alkylation of aldehydes [37]. This seminal work demonstrated that radical reactivity could be effectively merged with enamine organocatalytic activation to achieve not only high levels of enantioselectivity, but also paving the way to new avenues of performing radical reactions.

Over the last years, a wide range of organocatalytic and enantioselective radical transformations have been developed, with diarylprolinol silyl ether catalysts playing a central role [38]. The unique properties of this class of organocatalysts emerge from their versatility in enabling different photochemical activation modes depending on the decoration of the diarylprolinol silyl ether scaffold. In the following we will unfold these different activation modes and their applications.

**SCHEME 1:**
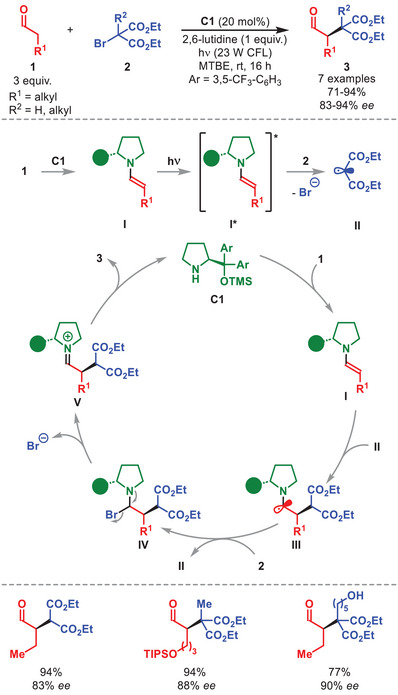
Photochemical α‐alkylation of aldehydes **1** with bromo‐malonates **2**.

In 2015 Melchiorre et al. introduced an innovative concept concerning the exploitation of the redox properties of organocatalytic enamines in their excited state (Scheme 1) [39]. Upon visible light excitation, enamine I*, formed from aldehyde **1** and diarylprolinol silyl ether **C1**, becomes a potent photoreductant, capable of generating radical **II** and bromide, through reductive cleavage of the C─Br bond in bromomalonate **2**. Next, another equivalent of ground state enamine **I** can trap **II** in a stereoselectively fashion, affording α‐amino radical **III**. Later mechanistic investigations revealed that the reaction proceeds through a radical chain propagation pathway [40]. This pathway involves an atom transfer radical addition in which **III** abstracts a bromine atom from **2**, thereby generating radical **II** and producing α‐bromo amine intermediate **IV**. Elimination of bromide affords the iminium‐ion intermediate **V** which, by hydrolysis, furnished the α‐alkylated aldehydes **3**.

The authors demonstrated the generality of the transformation providing a series of α‐alkylated aldehydes **3** in high yields and enantioselectivity (Scheme 1). Both secondary and tertiary bromomalonates **2** proved viable radical precursors, as well as different substituent patterns in **1**. It should also be noted that this strategy was extended to achieve the enantioselective γ‐functionalization of α,β‐unsaturated aldehydes via dienamine catalysis, and, later, to the development of a photocatalytic α‐sulfenylation of aldehydes applying **C1** as catalyst [41].

**SCHEME 2:**
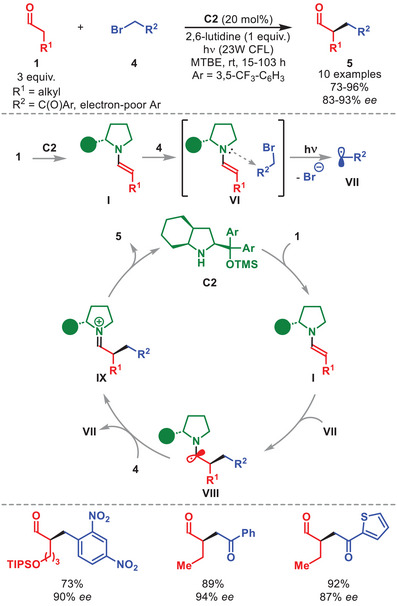
Photochemical α‐alkylation of aldehydes **1** with alkyl bromides **4**.

The Melchiorre group also established the first light‐driven radical method based on diarylprolinol silyl ether scaffold within the chemistry of electron donor–acceptor (EDA) complexes (Scheme 2) [42]. When an electron‐deficient alkyl bromide **4** was combined with a chiral enamine **I** generated from catalyst **C2**, the initial colorless reaction mixture turned bright yellow. This observation was attributed to the generation of an EDA complex **VI**, arising from orbitals sharing between the enamine **I** and **4**. A key feature of this complex is a shift of transfer‐band absorption to a longer wavelength, enabling excitation using visible light.

Upon excitation, the EDA complex **VI** undergoes single‐electron transfer (SET) leading to the generation of a radical anion, which then evolves into radical **VII** (Scheme 2). Another molecule of enamine **I** is trapped in a stereoselectively manner by **VII**, leading to α‐amino radical **VIII**. The authors demonstrated the reaction proceeds through a chain propagation mechanism, where **VIII** is oxidized by another molecule of alkyl bromide radical precursor **4**. Hydrolysis of **IX** afforded enantioenriched α‐alkylated aldehydes **5**. The method displayed a scope tolerating strongly electron‐deficient benzyl bromides and a wide set of phenacyl bromides **4** reacting with various aldehydes **1** and furnishing **5** in high yields and enantioselectivities. Notably, the strategy also enabled transformations of α‐phenylpentenal as dienamine precursors.

**SCHEME 3:**
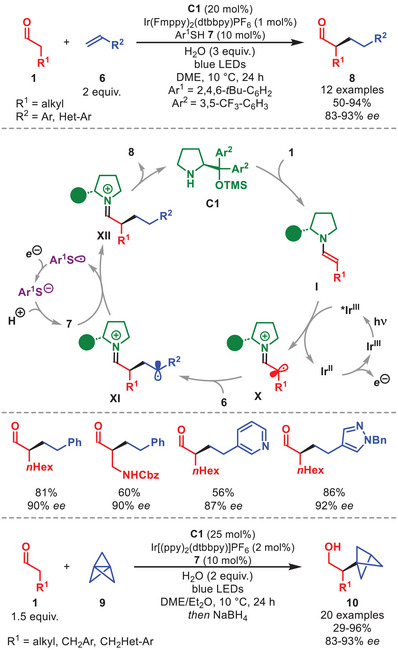
Top: Photochemical α‐alkylation of aldehydes **1** with styrenes **6**. Bottom: Photochemical α‐alkylation of aldehydes **1** with [1,1,1]propellane **9**.

An alternative approach integrates the oxidation of diarylprolinol silyl ether‐derived enamines by an external photocatalyst, as shown by MacMillan for the α‐alkylation of aldehydes **1** with styrenes **6** (Scheme 3) [43].

A series of α‐alkylated aldehydes **8** was obtained in good to high yields and excellent enantioselectivity across a broad range of reaction partners, including β‐ substituted and β,β‐disubstituted aldehydes, electron‐rich‐ and electron‐poor styrenes, as well as heteroaryl olefins (Scheme 3). An intramolecular variant of the transformation was also reported, enabled by an imidazolidinone‐type organocatalyst. The authors utilized a triple catalytic process involving organocatalyst **C1**, an iridium photocatalyst and a thiophenol‐based hydrogen atom transfer (HAT) catalyst **7**. The enamine **I** is oxidized by the photoexcited *Ir(III) to generating a α‐iminyl radical cation **X**, which then undergoes addition to **6** to form radical intermediate **XI**. A reductive HAT of this radical affords, after hydrolysis of iminium ion **XII**, the enantioenriched α‐alkylated aldehyde **8** while liberating **C1**. The catalytic cycle is closed by oxidation of the photocatalyst by the thiyl radical derived from **7**.

Later, Anderson et al. applied this concept to the enantioselective synthesis of α‐chiral bicyclo[1.1.1]pentanes **10** from aldehydes **1** and [1,1,1]propellane **9** (Scheme 3, bottom) [44].

**SCHEME 4:**
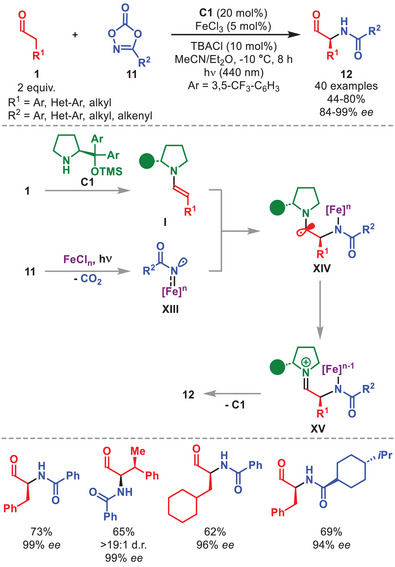
Photochemical α‐amidation of aldehydes **1** with dioxazolones **11**.

The ability of organocatalytic enamines to trap radicals has recently been exploited by Chang et al. in the development of an enantioselective α‐amidation of aldehydes using the synergistic action of diarylprolinol silyl ether **C1** and FeCl_3_ as catalysts (Scheme 4) [45].

The key step involves a visible‐light driven ligand‐to‐metal charge transfer to generate the active Fe(II)Cl_3_^−^ species from Fe(III)Cl_4_^−^, which activates dioxazolones **11** to form an iron‐acylnitrenoid radical **XIII** via a decarboxylative process (Scheme 4). This radical is stereoselectively trapped by the in situ generated enamine **I**, to give the α‐amino radical intermediate **XIV**. An intramolecular oxidative SET affords iminium ion **XV**, which upon hydrolysis furnishes α‐amido aldeydes **12**, regenerating both the organo‐ and metal‐based catalysts. The method displayed a broad scope, efficiently delivering a wide set of **12** with excellent enantioselectivity, employing aliphatic, aromatic, and heteroaryl‐substituted aldehydes and dioxazolones. Notably, the protocol is amenable to the late‐stage functionalization of aldehydes derived from drugs such as chlorambucil, oxaprozin, and D‐α‐tocopherol succinate.

**SCHEME 5:**
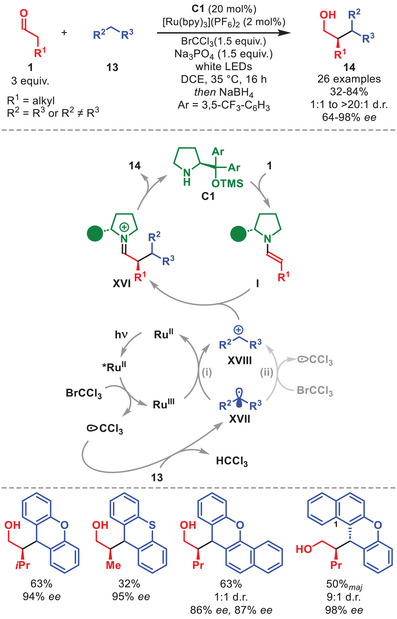
Photochemical α‐alkylation of aldehydes **1** with xanthenes **13**.

In 2017, Pericàs et al. exploited visible‐light photoredox catalysis in an enantioselective cross‐dehydrogenative coupling of aldehydes **1** with xanthenes **13**, using diarylprolinol silyl ether **C1** (Scheme 5) [46].

An array of alkylated alcohols (after reduction) **14**, bearing one or two stereocenters, was obtained in moderate to high yields and with excellent enantioselectivity using a range of aliphatic aldehydes, as well as substituted xanthenes (or thioxanthene), including benzo‐fused ones (Scheme 5). Diastereoselectivity was generally higher for xanthenes substituted at the C1‐position. Experiments and DFT studies revealed a dual catalytic mechanism, composed of photoredox‐ and organocatalytic cycles. The Ru‐photocatalyst is oxidatively quenched by BrCCl_3_, generating a CCl_3_ radical, which engages in the rate‐determining HAT with **13** affording radical **XVII**. The calculations indicated that the addition of carbocation **XVIII** to enamine **I**, leading to iminium ion **XVI**, is energetically more favorable than the corresponding pathway involving radical **XVII**. The authors proposed that the oxidation enabling the radical‐polar crossover might occur through reduction of Ru(III), thereby closing the photoredox cycle (i), although a radical chain process in which **XVII** is directly oxidized by another equivalent of BrCCl_3_ could not be excluded (ii). Finally, hydrolysis of **XVI** furnishes the enantioenriched **14**, regenerating **C1**.

**SCHEME 6:**
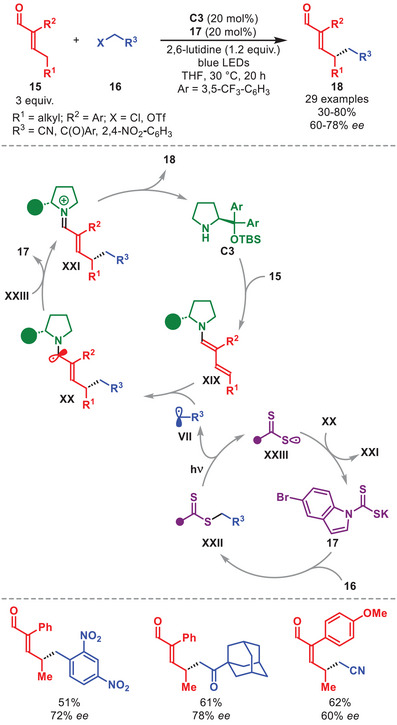
Photochemical γ‐alkylation of α,β‐unsaturated aldehydes **15** with alkyl halides **16**.

Beyond the ability of the excited state organocatalytic dienamines to act as a photoreductants to bromomalonates or engage in EDA with electron‐poor benzyl or phenacyl bromides [41, 42], Mechiorre et al. in 2022 disclosed a general enantioselective γ‐alkylation of α,β‐unsaturated aldehydes **15** by merging dienamine‐ and dithiocarbamate (DTC) catalysis [47], using difficult‐to‐reduce alkyl chlorides **16** as radical precursors (Scheme 6) [48]. A library of γ‐alkylated enals **18** was obtained in good yields and enantioselectivities using phenacyl chloride, aliphatic α‐chloro ketones, and benzyl chlorides as radical precursors **16**, and **15** embedding different substituents with various electronic‐ and steric properties. Quantum‐yield measurements supported a closed catalytic cycle in which diarylprolinol silyl ether **C3** and an indole‐based DTC catalyst **17** cooperate to form enantioenriched **18**. The photoactive intermediate **XXII** is generated through an S_N_2 substitution of **16** by catalyst **17**. Blue‐light excitation of **XXII** triggers homolytic cleavage, furnishing the thiyl‐radical **XXIII** and the carbon‐centered radical **VII**. This radical is then enantioselectively trapped by dienamine **XIX**, generated by condensation of **15** with **C3**, exclusively at the remote γ‐carbon. Turnover of **17** is given by a single‐electron oxidation of the resulting α‐amino radical **XX**. Finally, hydrolysis of iminium ion **XXI** delivers **18** and regenerates catalyst **C3**. Interestingly, the use of linear pentenal or hepta‐2,4‐dienal (as trienamine precursor) afforded exclusively γ‐ or ε‐functionalized products, respectively, even though as racemates. It is worth mentioning that the same group combined DTC catalysis with enamine catalysis for the enantioselective α‐alkylation of aldehydes using diarylprolinol silyl ether as catalyst [47].

**SCHEME 7:**
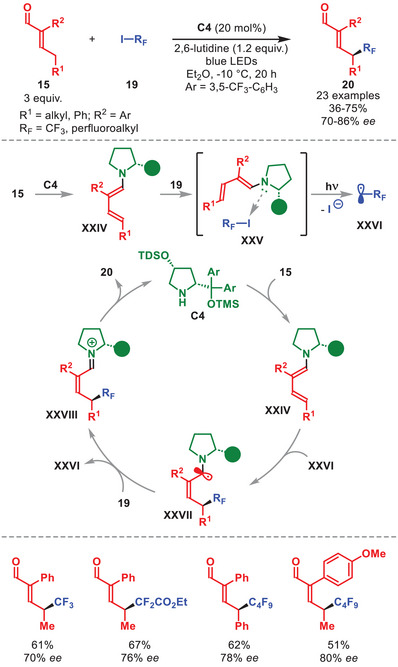
Photochemical γ‐perfluoroalkylation of α,β‐unsaturated aldehydes **15** with alkyl halides **19**.

Later, the same group reported a photochemical, enantioselective γ‐perfluoroalkylation of α‐branched α,β‐unsaturated aldehydes **15** (Scheme 7) [49]. The use of chiral diarylprolinol silyl ether **C4** generates the corresponding dienamine **XXIV**, which forms photoactive EDA complex **XXV** with perfluoroalkyl iodide **19**. Under blue light irradiation, this complex undergoes SET affording radical **XXVI**, which is trapped by another molecule of **XXIV** in a regio‐ and stereoselective fashion. Quantum yield measurement indicated the operation of a radical chain process, in which the ensuing α‐amino radical **XXVII** regenerates radical **XXVI** via a SET event, or through abstraction of an iodine atom from **19** via an atom transfer radical addition. Final hydrolysis of the iminium ion **XXVIII** affords the γ‐perfluoroalkyled enal **20**, while regenerating **C4**. The generality of this γ‐regioselective perfluoroalkylation was demonstrated by the formation of **20** in moderate to high yields and generally good enantioselectivities, using α‐aryl **15** and **16** of different perfluoroalkyl chain lengths, including CF_3_, α‐difluoro esters, and sulfonyl fluorides.

**SCHEME 8:**
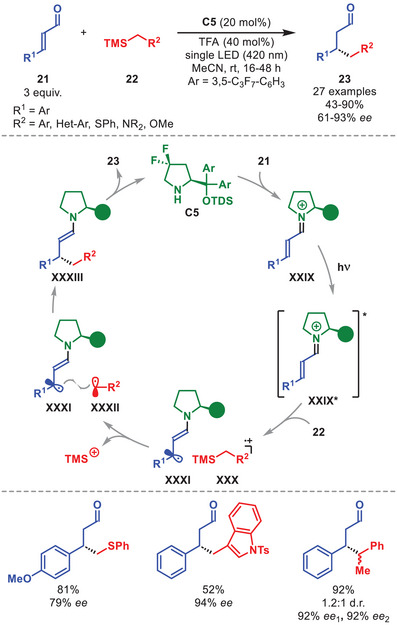
Photochemical β‐alkylation of α,β‐unsaturated aldehydes **21** with benzyl silane **22**.

Building on their earlier discovery that organocatalytic enamines can operate as strong reductants in their photoexcited state, the Melchiorre group proposed that photoexcited iminium ion XXIX* could instead serve as a potent oxidant, enabling single‐electron oxidation of electron‐rich benzyl silane **22** to generate reactive open‐shell intermediates. This concept was applied to develop an enantioselective radical β‐benzylation of α,β‐unsaturated aldehydes **21** (Scheme 8) [50]. Condensation of diarylprolinol silyl ether **C5** with **21** generates iminium ion **XXIX** which can absorb light reaching its excited state XXIX*. Single‐electron oxidation of **22** by XXIX* enables generation of radical cation **XXX**, which spontaneously releases trimethylsilyl cation. The resulting radical **XXXII** undergoes a stereocontrolled radical–radical coupling with the chiral β‐enaminyl radical **XXXI**, affording enamine **XXXIII**. This species, upon isomerization and hydrolysis, furnishes mainly β‐benzylated aldehydes **23**, while regenerating catalyst **C5**. A characteristic feature of this study is the catalyst design: installation of *gem*‐difluoride substituents at the C4‐position of the diarylprolinol silyl ether scaffold increases its oxidation potential, preventing catalyst degradation by the excited iminium ion and thereby enabling higher efficiency, while increasing enantioselectivity by utilizing the bulkier TDS (thexyldimethylsilyl) group.

This strategy allowed for access to β‐functionalized aldehydes **23** in moderate to good yields and high enantioselectivities using an array of cinnamaldehydes and benzyl‐ or heteroaryl‐silanes, including a product with two vicinal stereocenters, even though with low diastereoselectivity (Scheme 8). Furthermore, α‐silyl thioethers, α‐silyl amines, and α‐silyl ethers were also found to engage in the photochemical reaction with comparable results. The strategy was subsequently extended to enantioselective biradical coupling‐based β‐functionalizations of iminium ions using diarylprolinol silyl ether catalysts [51, 52, 53, 54, 55, 56].

**SCHEME 9:**
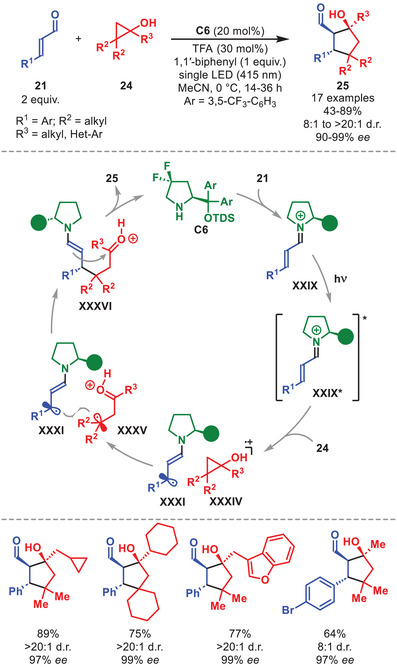
Photochemical annulation between α,β‐unsaturated aldehydes **21** and cyclopropanols **24**.

An example involving a photochemical cascade process that merges the photoexcitation of iminium ions with the ground‐state reactivity of enamines should be mentioned [53]. This directly converts α,β‐unsaturated aldehydes **21** and cyclopropanols **24** into cyclopentanols **25** in good yields and with excellent stereoselectivity in the presence of diarylprolinol silyl ether **C6** (Scheme 9). Cyclopropanols **24** with linear or branched aliphatic‐, benzylic‐, and heterocyclic substituents, as well as spirocyclic variants, were well tolerated. Diverse β‐aryl substitution patterns were amenable within **21**, while β‐alkyl fragments inhibited the reaction. The authors proposed a catalytic cycle where **24** undergoes SET oxidation by the excited iminium ion XXIX* to form unstable oxycyclopropyl radical cation **XXXIV**, which rapidly ring‐opens to give **XXXV**. This species engages in a stereoselective radical–radical coupling with the β‐enaminyl radical **XXXI**, furnishing the ground‐state enamine **XXXVI**, which subsequently is involved in an intramolecular aldol cyclization to deliver the highly diastereo‐ and enantioenriched **25** bearing three contiguous stereocenters.

**SCHEME 10:**
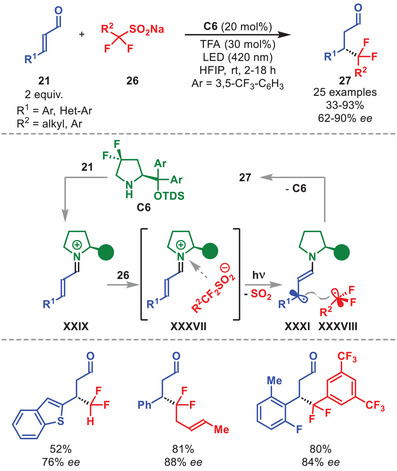
Photochemical β‐difluoroalkylation of α,β‐unsaturated aldehydes **21** with sulfinates **26**.

In 2022, Alemán et al. disclosed an alternative strategy to generate β‐enaminyl radicals from organocatalytic iminium‐ion intermediates. They demonstrated that the electron–donor character of *gem*‐difluoride sulfinates **26** enables formation of an EDA complex **XXXVII** with iminium ion **XXIX**, obtained from condensation of diarylprolinol silyl ether **C6** with α,β‐unsaturated aldehydes **21**, thereby allowing for an enantioselective β‐difluoroalkylation of **21** (Scheme 10) [57]. Under visible‐light irradiation, excitation of complex **XXXVII** triggers a SET event that simultaneously furnishes β‐enaminyl radical **XXXI** and a *gem*‐difluoride radical **XXXVIII** (after SO_2_ extrusion). A subsequent stereoselective radical–radical coupling followed by catalyst‐regenerating hydrolysis affords the β‐difluoroalkylated aldehydes **27**.

The applicability of the protocol was ascertained using a number of different substrates allowing for the access to β‐difluoroalkylated aldehydes **27** in moderate to high yields and generally high enantioselectivities (Scheme 10). First, various substitution patterns in the aromatic or hetero‐aromatic moiety of α,β‐unsaturated aldehydes **21** proved viable. The successful utilization of non‐substituted, aryl‐, heteroaryl‐, and CF_2_‐alkyl sulfinates **26** further expanded the generality of the protocol.

More recently, another example of an EDA complex derived from an iminium ion of type **XXIX** enabling enantioselective β‐functionalization of α,β‐unsaturated aldehydes was reported by Quintavalla et al. [58].

**SCHEME 11:**
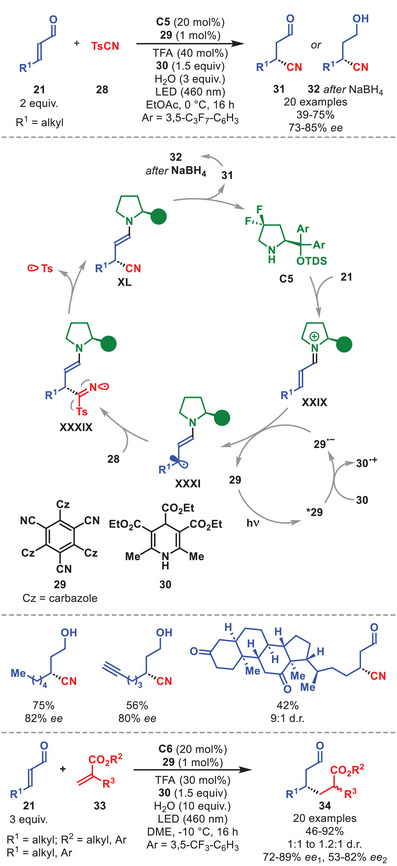
Top: Photochemical β‐cyanation of α,β‐unsaturated aldehydes **21** with TsCN **28**. Bottom: Photochemical β‐alkylation of α,β‐unsaturated aldehydes **21** with acrylates **33**.

The use of electron‐rich β‐enaminyl radicals **XXXI** proved crucial for addressing the long‐standing challenge of elusive stereoselective conjugate cyanation of α,β‐unsaturated aldehydes **21** (Scheme 11). A stereoselective method, in which the cooperative action of diarylprolinol silyl ether **C5** and a visible‐light‐activated photoredox catalyst **29** was disclosed, enabling this unprecedented disconnection with β‐enaminyl radical **XXXI** being generated by reduction of the corresponding iminium ion **XXIX** by the external photocatalyst [59]. Upon excitation, photocatalyst **29** is quenched by dihydropyridine **30**, affording **29^•–^**, which then reduces iminium ion **XXIX** (formed by condensation of diarylprolinol silyl ether **C5** and α,β‐unsaturated aldehyde **21**) to deliver the chiral β‐enaminyl radical **XXXI**. This transient species intercepts TsCN **28** with high regio‐ and stereoselectivity. The ensuing radical **XXXIX** undergoes β‐fragmentation to form enamine **XL**, while releasing a tosyl radical. Hydrolysis of **XL** provides β‐cyanoaldehydes **31**, regenerating catalyst **C5**.

The method tolerates a range of aliphatic β‐substituents on α,β‐unsaturated aldehydes **21**, including branched or alkene‐/alkyne‐tethered chains, as well as β‐aryl groups, efficiently affording β‐cyanoaldehydes **31** with full β‐regioselectivity and high enantioselectivity (Scheme 11). Biorelevant residues can also be employed, as demonstrated by the reaction of an α,β‐unsaturated aldehydes derived from deoxycholic acid. Under slightly modified conditions, this strategy could also be applied to the stereoselective β‐addition of acrylates **33**, affording the corresponding β‐alkylated aldehydes **34** in good to high yields and enantioselectivity, albeit with modest diastereocontrol (Scheme 11, bottom).

**SCHEME 12:**
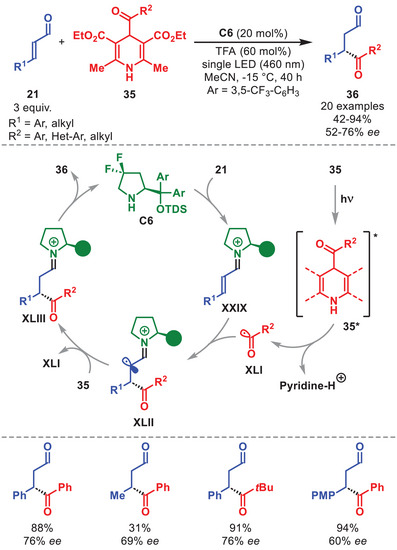
Photochemical β‐acylation of α,β‐unsaturated aldehydes **21** with dihydropyridines **35**.

The organocatalytic generated iminium ion **XXIX**, derived from diarylprolinol silyl ether **C6**, can participate in Giese‐type acyl‐radical additions, enabling the synthesis of chiral 1,4‐dicarbonyl compounds **36** as presented by Melchiorre et al. in 2019 (Scheme 12) [60].

Visible‐light excitation of 4‐acyl‐1,4‐dihydropyridine **35** generates acyl radical **XLI**, which is intercepted by the ground‐state iminium ion **XXIX**. The resulting intermediate **XLII** undergoes a HAT with a second molecule of **35**, furnishing iminium ion **XLIII**. Hydrolysis of **XLIII** then delivers the enantioenriched 1,4‐dicarbonyl **36** and regenerates catalyst **C6**. The scope of the acyl radical conjugate addition tolerates a variety of radical precursors **35** embedding aromatic, heteroaryl, and alkyl groups, efficiently furnishing **36** with moderate to good enantioselectivity. Variously β‐aryl‐ and β‐alkyl‐substituted α,β‐unsaturated aldehydes **21** also proved suitable.

Independently, Yu et al. reported a similar transformation employing catalyst **C6** along with a Ru‐photocatalyst and α‐ketoacids as acyl radical precursors [61]. The utility of the iminium ion as radical traps in enantioselective Giese‐type additions was expanded by the Melchiorre group, particularly in combination with an external acridinium‐based photocatalyst, to achieve the enantioselective β‐alkylation of β‐aryl‐ or β‐alkyl‐substituted α,β‐unsaturated aldehydes [62], and the stereoselective synthesis of 1,7‐dicarbonyl compounds [63].

**SCHEME 13:**
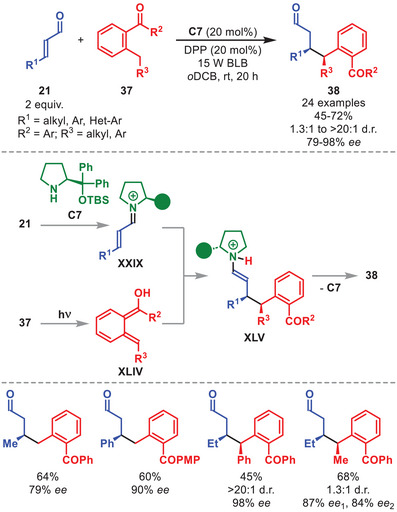
Photochemical β‐benzylation of α,β‐unsaturated aldehydes **21** with benzophenones **37**. DPP = diphenylphosphoric acid.

In 2017, Melchiorre et al. reported a light‐driven β‐benzylation of α,β‐unsaturated aldehydes **21** (Scheme 13) [64]. Irradiation of 2‐alkyl benzophenones **37** with 365 nm light triggers a 1,5‐HAT event to generate *ortho*‐quinomethane photoenol **XLIV**, which undergoes a vinylogous Michael addition to iminium ion **XXIX** to furnish intermediate **XLV**. While the use of diphenylphosporic acid as co‐catalyst was required to improve the conversion of the transformation, utilization of the diarylprolinol silyl ether **C7** provided the β‐benzylated aldehydes **38** in moderate to good yields and with high enantioselectivity. Several β‐alkyl‐ and β‐aryl substituted **21** proved viable, as well as acyclic‐ and cyclic **37**, adorned with various substituent patterns.

**SCHEME 14:**
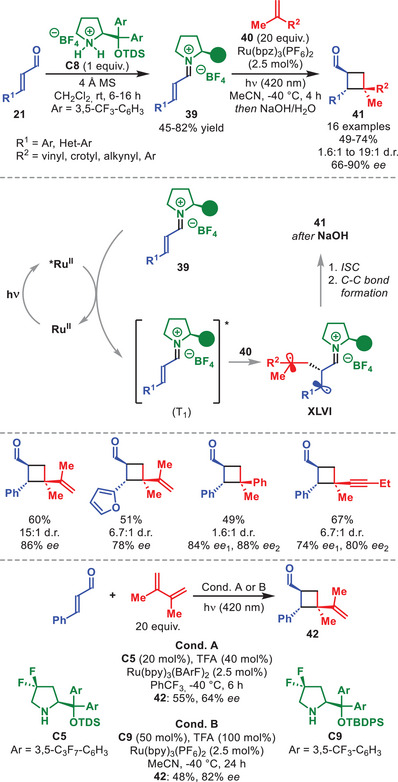
Top: Photochemical [2+2] cycloaddition between stochiometric iminium ions **39** and olefins **40**. Bottom: Catalytic variant.

As shown by some of the examples discussed above, the photochemistry of iminium ions generated from α,β‐unsaturated aldehydes and diarylprolinol silyl ether catalysts has enabled a wide range of enantioselective transformations. These processes rely on the singlet excited state (S_1_) of the iminium‐ion intermediate. In 2020, Bach et al. enabled the triplet excited state (T_1_) reactivity of stochiometric chiral iminium ion salts **39**, prepared from α,β‐unsaturated aldehydes **21** and diarylprolinol silyl ether salt **C8**, in a Ru‐catalyzed, intermolecular [2+2] photocycloaddition with olefins **40** to afford cyclobutanes **41** with moderate to high diasteroselectivity and high enantioselectivity after alkaline hydrolysis (Scheme 14) [65].

The reaction proved compatibility with various iminium ions **39**, tolerating diversely substituted aryl groups, as well as a number of conjugated olefins **40** (Scheme 14). Mechanistic studies revealed that visible‐light excitation of the Ru‐photocatalyst triggers a triplet‐energy transfer to the iminium salt, which then reacts with the olefin to form the 1,4‐diradical intermediate **XLVI**. After intersystem crossing (ISC), this intermediate undergoes the subsequent C–C bond‐forming step. Despite the inherent challenges posed by this system, careful optimization of the reaction conditions enabled the authors to develop two catalytic variants of the transformation involving cinnamaldehyde and dimethyl butadiene (utilizing catalysts **C5** or **C9**), which furnished cyclobutane **42** in moderate yield and with good enantioselectivity (Scheme 14, bottom).

To summarize, the photochemical reactivity of enamines and iminium ions generated from diarylprolinol silyl ether catalyst can be rationalized as follows. In the first scenario, a photoinduced SET involves the organocatalytic intermediate, either as an enamine or an iminium ion, enabling radical generation by direct excitation or EDA‐complex formation/excitation. In a complementary mode, light is used to generate radical species from external precursors through a direct excitation manifold or by mediation of a metal‐ or organic‐based photocatalyst or a DTC catalyst. These radicals are then typically trapped by ground‐state enamines or iminium ions in stereoselective C─C bond‐forming events, typically following a Giese‐type reaction, or engaged in radical–radical couplings with α‐iminyl radical cations (from enamines) or β‐enaminyl radicals (from iminium ions). Finally, less‐common activation pathways have also been disclosed, including energy‐transfer activation of stochiometric iminium ions to access triplet‐state reactivity, as well as polar photochemical processes in which light serves to generate electrophilic or nucleophilic partners that react with organocatalytic intermediates following a two‐electron‐logic pathway.

## 3 Electrochemical Transformations

Recent years have witnessed a renaissance of organic electrosynthesis as a sustainable strategy for constructing complex molecular architectures [66, 67]. Within this landscape, enantioselective electrochemical methods have emerged as increasingly powerful tools. Although electrochemistry has already demonstrated successful synergy with asymmetric metal catalysis [68] and biocatalysis [69], its combination with asymmetric aminocatalysis is still in its infancy [70, 71].

In 2009 the Jang's group reported a seminal enantioselective radical α‐oxamination of aldehydes, in which a radical–radical coupling between TEMPO and a α‐iminyl radical cation—generated from electrochemical oxidation of an organocatalytic enamine—is the key step [72]. The following year, our group introduced a distinct concept, demonstrating that electricity could be used to generate a transient electrophilic Michael acceptor through the anodic oxidation of a tosyl *para*‐hydroxy aniline, which then reacted stereoselectively with an organocatalytic enamine [73]. Notably, in both seminal studies, the enamine intermediates were derived from diarylprolinol silyl ether catalysts. Although several reports over the past decade have merged electrochemistry with enamine catalysis [74], it is only in the last couple of years that diarylprolinol silyl ether catalysts have been strategically applied to these systems to advance the seminal concepts.

**SCHEME 15:**
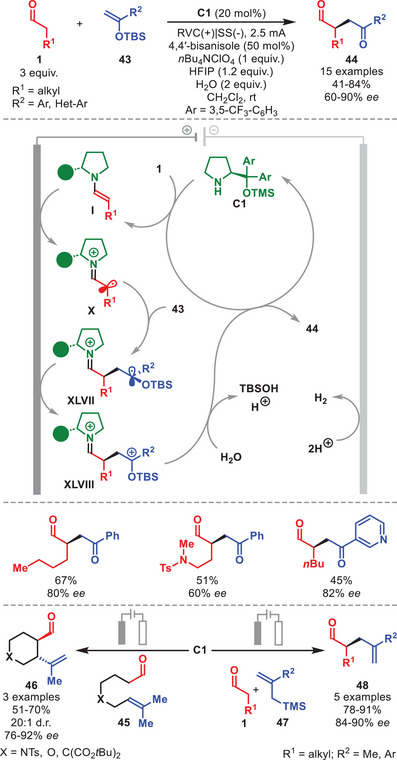
Top: Electrochemical α‐alkylation of aldehydes **1** with silyl enol ethers **43**. Bottom: α‐allylation of aldehydes **1** with allyl silanes **47** and intramolecular variant.

In 2024, Dell'Amico et al. reported an enantioselective electrochemical α‐alkylation of aldehydes **1**, exploiting the anodic oxidation of enamine **I** generated from catalyst **C1** (Scheme 15) [75].

The enamine **I** is oxidized at the anode to form the corresponding α‐iminyl radical cation **X**, which is then stereoselectively trapped by silylenol ether **43** to furnish radical cation **XLVII** (Scheme 15). A subsequent oxidation step affords intermediate **XLVIII**, whose hydrolysis delivers α‐alkylated aldehydes **44**. The electrochemical circuit is closed by cathodic hydrogen evolution. Mechanistic experiments and DFT calculations indicated the importance of 4,4′‐bisanisole acting as a radical shuttle, mitigating the rise of the cell potential over time, thereby preventing decomposition of **C1**, while simultaneously facilitating the oxidation of enamine **I** to **X**. This electrochemical method proved tolerant to variation of both substrates, enabling access to a panel of **44** in moderate to high yields and generally with high enantioselectivity. The authors extended the methodology to an enantioselective α‐allylation of aldehydes using allyl silanes **47**. This transformation was adapted to an intramolecular variant using aldehydes **45**, providing cycloadducts **46** with excellent diastereoselectivity and high enantioselectivity.

**SCHEME 16:**
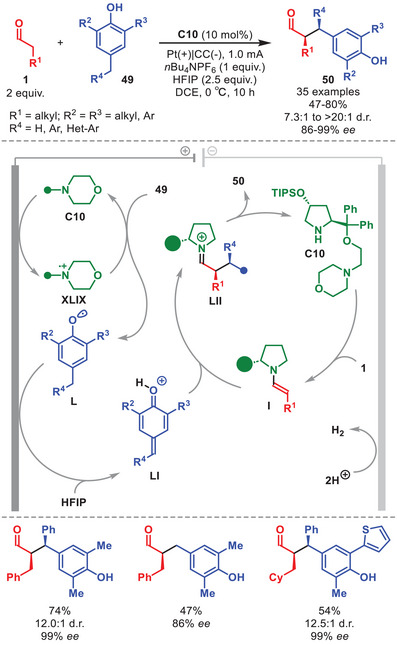
Electrochemical α‐alkylation of aldehydes **1** with phenols **49**.

An example of electrochemical in situ generation of electrophiles was accomplished in the same year by Xu et al., that designed an ad hoc bifunctional catalyst **C10** to enable a stereoselective α‐alkylation of aldehydes **1** with phenols **49** (Scheme 16) [76].

The novelty lay in the strategic incorporation of a morpholine redox‐mediator moiety into the diarylprolinol scaffold. Catalyst **C10** thus covered a double function in the system: it generates the reactive enamine **I** while acting as a redox mediator (Scheme 16). Indeed, the anodically oxidized form of **C10** (**XLIX**) efficiently promotes the oxidation of phenol **49** to radical **L**, which is further oxidized at the anode to generate the electrophilic *para*‐quinone methide **LI**. This species is engaged in a stereoselective Michael addition with enamine **I**, furnishing iminium ion **LII**, which upon hydrolysis yields the α‐alkylated aldehyde **50**. The addition of HFIP proved crucial, both for activating intermediate **LI** and for increasing the diastereoselectivity by improving face selectivity of the catalyst in the transition state. The authors experimentally confirmed that using the two components separately—that is, a simplified C4‐substituted diarylprolinol silyl ether catalyst and *N*‐methyl morpholine—resulted in much lower yield. The scope of the transformation was broad, providing **50** in good yields and with excellent diastereo‐ and enantiocontrol. Notably, both symmetrical and non‐symmetrical 2,4,6‐substituted phenols **49** were suitable, as well as aldehydes derived from biorelevant scaffolds such as pregabalin and estrone. Although this transformation could be achieved under classical polar conditions in comparable efficiency and stereoselectivity, as previously reported [77], this electrochemical approach circumvents the need to synthesize difficult‐to‐make *para*‐quinone methides **LI**.

**SCHEME 17:**
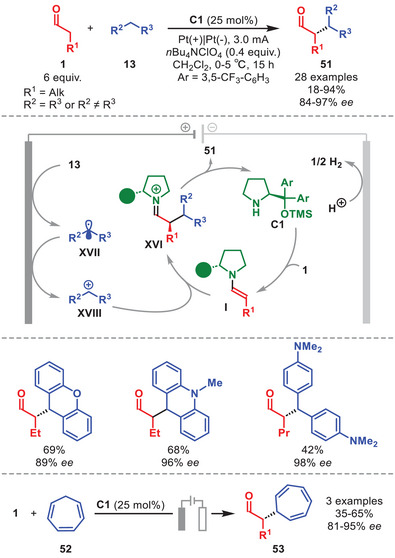
Top: Electrochemical α‐alkylation of aldehydes **1** dibenzylic substrates **13**. Bottom: Electrochemical α‐alkylation of aldehydes **1** with cycloheptatriene **52**.

Concomitantly, the group of Pan developed an enantioselective electrochemical cross‐dehydrogenative coupling reaction between dibenzylic substrates **13** and aldehydes **1** using catalyst **C1** (Scheme 17) [78]. Cyclic voltammetry and radical‐clock experiments support a mechanism initiated by the anodic oxidation of **13** to generate radical **XVII**. Analogously to the photochemical variant previously reported by Pericàs [45], the corresponding dibenzylic electrophilic cation **XVIII** arises from a radical‐polar crossover step. In this case, the oxidation of radical **XVII** to **XVIII** occurs at the Pt‐anode. This carbocation is then enantioselectively intercepted at the α‐position of enamine **I**, thereby delivering α‐alkylated aldehyde **51**, after hydrolysis of iminium ion **XVI**, in variable yields and with high diastereo‐ and enantioselectivitity. The methodology exhibited broad applicability and excellent stereocontrol, tolerating a range of radical precursors such as xanthenes, methylacridines, and electron‐rich diarylmethanes. A variety of aldehydes, including the naturally occurring citronellal, were also compatible. The authors further demonstrated the electrochemical coupling of **1** with cycloheptatriene **52**, providing the corresponding chiral adducts **53** in high enantioselectivity (Scheme 17, bottom).

**SCHEME 18:**
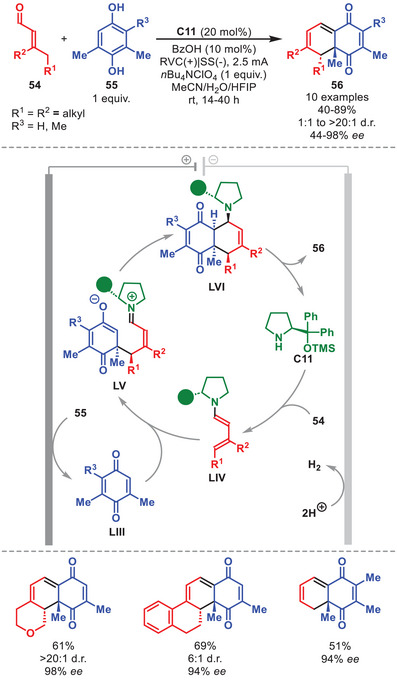
Electrochemical [4+2] cycloadditions between α,β‐unsaturated aldehydes **54** and hydroquinones **55**.

The combination of electrochemistry with aminocatalytic intermediates can also be extended to dienamine catalysis, as recently demonstrated by Albrecht et al. in the development of an eliminative, enantioselective Diels–Alder reaction between hydroquinones **55** and α,β‐unsaturated aldehydes **54**, in the presence of catalyst **C11** (Scheme 18) [79]. Although the scope of hydroquinone **55** proved relatively narrow, a range of cyclic and acyclic **54** bearing diverse substitution patterns were well tolerated, thereby enabling access to polycyclic adducts **56** featuring different ring‐fusions in moderate to high yields and with high diastereo‐ and enentioselectivity. A sequential reaction pathway was proposed, in which an initial anodic oxidation of **55** generates the corresponding electrophilic quinone **LIII**, which then undergoes a stereoselective Michael addition at the γ‐position of the organocatalytically generated dienamine **LIV**, forming zwitterionic intermediate **LV**. Subsequent intramolecular cyclization affords **LVI**, which readily eliminates the catalyst furnishing cycloadduct **56**. Based on previous DFT studies of a previous related process [80], it was suggested that this transformation proceeds via an *endo*‐selective, step‐wise cycloaddition.

**SCHEME 19:**
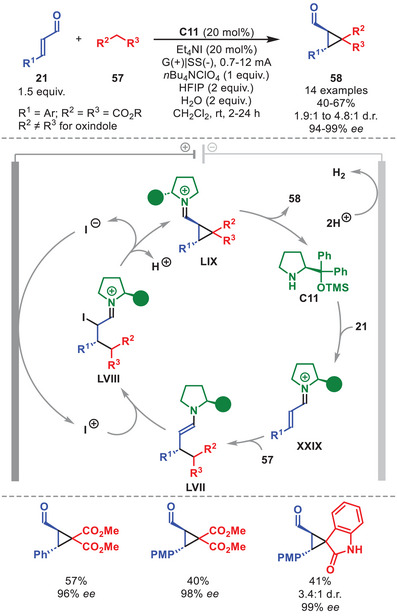
Electrochemical cyclopropanation of α,β‐unsaturated aldehydes **21** with malonates or oxindoles **57**.

So far, a unique example involving an organocatalytic iminium‐ion intermediate in asymmetric electrochemistry was recently reported by Ošeka et al. (Scheme 19) [81]. It was shown that an electrochemical enantioselective cyclopropanation of α,β‐unsaturated aldehydes **21** with malonates or oxindoles **57** could be achieved through a **C11**‐promoted electro‐organocatalytic cascade process. A panel of cyclopropane‐carbaldehydes **58** was obtained in moderate to good yields, with generally good diastereoselectivity and excellent enantioselectivity. A range of β‐aryl‐substituted **21** proved suitable, as did various **57** as nucleophiles, thereby enabling access even to oxindole‐based enantioenriched spirocycles. The authors proposed that condensation of **C11** with **21** generates iminium ion **XXIX**, which first undergoes a stereoselective Michael addition with **57** to furnish enamine **LVII**. Concurrently, anodic oxidation of iodide forms electrophilic iodonium, that reacts at the α‐position of enamine **LVII**. The incipient iminium ion **LVIII** next undergoes an intramolecular nucleophilic substitution, regenerating iodide in the electrocatalytic cycle and forming the cyclopropane ring within **LIX**. Final hydrolysis releases **58**, while regenerating catalyst **C11**.

## 4 Dual Catalytic Transformations

In contrast to a catalytic system involving only a single catalyst to activate the nucleophile or electrophile in a single catalytic cycle, in synergistic dual catalysis two catalysts controlling two individual cycles work in synergy to form a new bond. Each of the two catalysts serve to activate the nucleophile and electrophile concurrently, thus reducing the HOMO–LUMO gap, enabling otherwise unfeasible or ineffective transformations [82, 83, 84]. The feasibility of combining aminocatalysis and transition metal catalysis was first demonstrated by the group of Córdova in 2006 when subjecting α‐enolizable aldehydes to allyl acetate in the presence of pyrrolidine and Pd(PPh_3_)_4_ to facilitate a racemic α‐allylation [85].

This work was later expanded by Carreira et al. in 2013 when disclosing the fully stereodivergent α‐allylation of α‐branched aldehydes with allylic alcohols by a synergistic iridium and primary cinchona alkaloid aminocatalyst system [86]. Thus, the aldehyde is activated by an aminocatalyst through enamine catalysis, while the allylic alcohol is concurrently activated by a chiral iridium catalyst to generate a π‐allyl complex, each controlling the configuration of one of the two stereocenters in the product. Shortly after, Carreira et al. expanded the methodology to function with diarylprolinol silyl ether catalysts for aldehydes that are not α‐branched [87, 88]. Since then, the field has expanded significantly with dual catalytic protocols involving the diarylprolinol silyl ether class of catalysts in concert with various transition metal‐ and Lewis acid‐based systems. This chapter will focus mostly on the application of transition metals and Lewis acids in concert with aminocatalysis to highlight recent key examples to access otherwise unattainable scaffolds.

**SCHEME 20:**
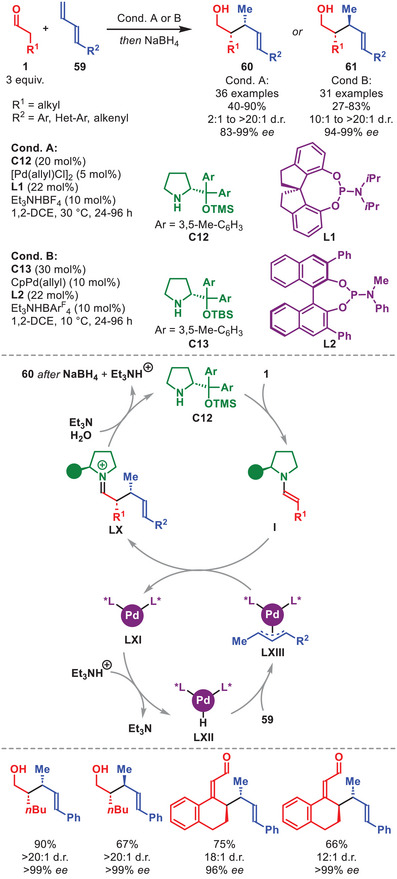
Pd‐catalyzed stereodivergent hydroalkylation of aldehydes **1** with 1,3‐dienes **59**.

The influence of Carreira's original system remains visible on the field to this day. One recent example was disclosed by the group of Zi involving the stereodivergent hydroalkylation of α‐enolizable aldehydes **1** with 1,3‐dienes **59** to access both the *syn*‐ and *anti‐*configured coupling products **60** and **61**, respectively, in moderate to high yield and overall good to excellent stereocontrol (Scheme 20) [89]. The protocol leading to the *syn*‐configured **60** was more selective than the *anti*‐protocol. The developed protocol is amenable to various aliphatic **1** and **59** carrying aromatic or olefinic substituents; however, aliphatic substituents were not tolerated. DFT calculations showed that the in situ generated chiral palladium complex **LXI** is first protonated to generate PdH species **LXII**, whose insertion into **59** from the *Si*‐face generates Pd‐π‐allyl complex **LXIII**. Stereoselective nucleophilic attack by enamine **I** affords iminium ion **LX** when **L1** was applied, which after hydrolysis of the catalyst provides **60**. The authors demonstrated that the protocol could even be applied to dienamine catalysis.

Multiple similar transformations hinging on chiral palladium‐ or iridium‐π‐allyl complexes in combination with the diarylprolinol silyl ether catalysts have been reported in the last decade, such as the α‐allylation of various uncommon enamine precursors [87, 90, 91, 92, 93], and in aminocatalytic cascade reactions [94, 95, 96]. It is worth noting that an α‐allylation without relying on the utilization of transition metals was accomplished by Hall et al. exploiting boronic acids in a synergistic system with aminocatalysis [97]. In this protocol the boronic acid serves to dehydrate the substrate allylic alcohol and generate a reactive carbocation intermediate capable of reacting with the in situ generated enamine.

**SCHEME 21:**
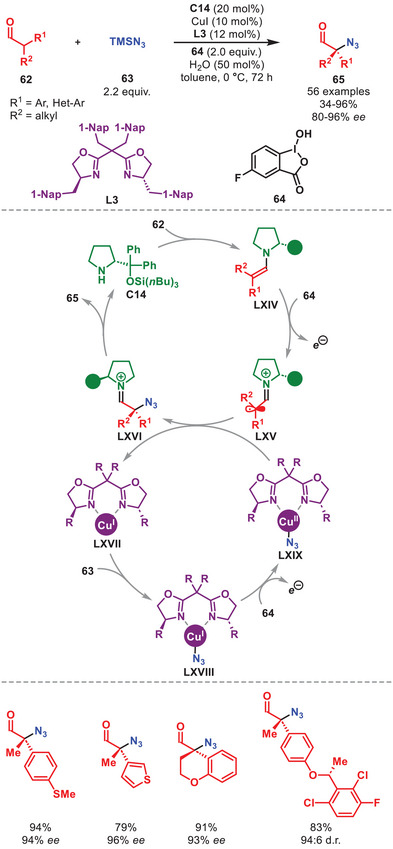
Cu‐catalyzed α‐azidation of α‐branched aldehydes **62** with TMSN_3_ **63**.

Despite the appearance of photochemical alternatives, aminocatalytic SOMO activation [98, 99] has recently been utilized in combination with a copper‐mediated enantioselective α‐azidation of α‐branched aldehydes **62** (Scheme 21) [100]. The method was amenable toward both cyclic‐ and acyclic α‐branched aldehydes carrying an aromatic moiety and afforded α‐azidoaldehydes **65** in moderate to high yields and high to excellent enantioselectivity. The catalytic cycle is initiated by the condensation of **C14** to **62** forming enamine **LXIV**, which after SET by the sacrificial oxidant **64** generates α‐iminyl radical cation **LXV**. Concomitantly, the Cu(I) complex **LXVII** interacts with TMSN_3_ **63** to generate **LXVIII**, which after SET affords the active Cu(II)‐N_3_ species **LXIX**. It was then proposed, that the open‐shell intermediate **LXV** can be sequestered by the Cu(II)‐N_3_ species **LXIX** via an outer‐sphere ligand transfer step to generate **LXVI**, and subsequently **65** after aminocatalyst hydrolysis. Intriguingly, the utilization of chirality pairing between the aminocatalyst and ligand is essential to achieving high enantioselectivity.

**SCHEME 22:**
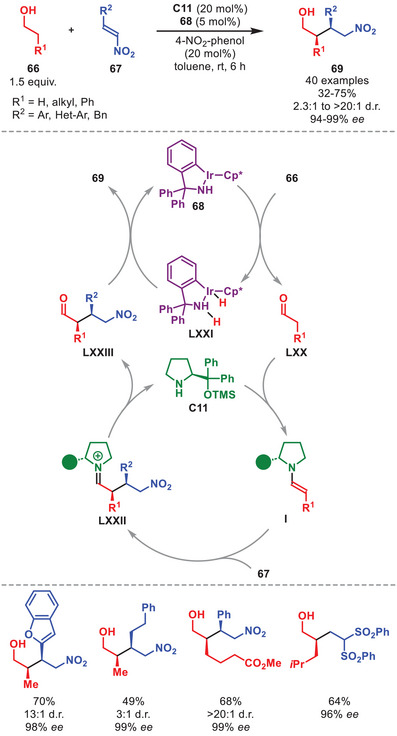
Ir‐catalyzed α‐functionalization of alcohols **66** with nitrosyrenes **67**.

The combination of aminocatalysis with redox‐active transition metals has enabled the α‐functionalization of alcohol substrates via a transient aldehyde intermediate through borrowing hydrogen relay catalysis [101]. This is shown in the work of Zhao et al. detailing the α‐alkylation of aliphatic and aromatic alcohols **66** by nitrostyrenes **67** catalyzed by diarylprolinol silyl ether **C11** and iridium complex **68** (Scheme 22) [102].

This protocol notably provided improved diastereocontrol compared to starting from the corresponding aldehydes (15:1 vs. 1.7:1 d.r.), hypothesized to originate from the suppression of the product aldehyde (**LXXIII**) epimerization, and enabled the direct functionalization of simple alcohols in overall good to excellent yield and selectivity (Scheme 22). The proposed mechanism commences by a two‐electron oxidation of alcohol **66** to generate aldehyde **LXX** by iridium complex **68** forming **LXXI**. After condensation of **C11** to generate enamine **I**, the stereoselective Michael addition to nitrostyrene **67** proceeds to generate iminium ion **LXXII**. After hydrolysis of the aminocatalyst and two‐electron reduction by **LXXI**, Michael adduct **69** is formed while regenerating both catalysts. The transformation was also feasible for vinyl sulfone electrophiles.

In the past decade borrowing‐hydrogen catalysis has been combined with aminocatalysis on multiple occasions primarily for the in situ generation of α,β‐unsaturated aldehydes from allylic alcohols engaged in iminium‐ion catalysis [103, 104, 105].

**SCHEME 23:**
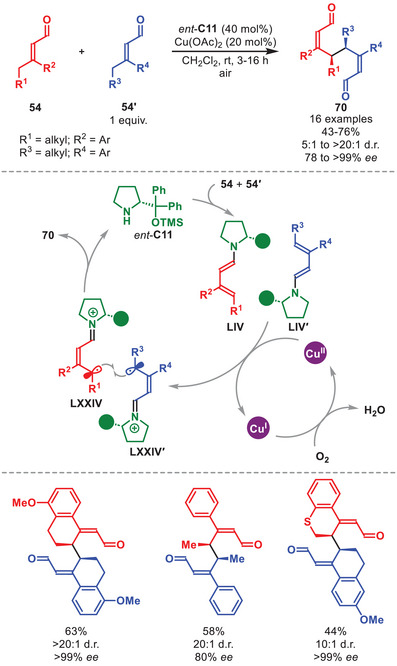
Cu‐catalyzed oxidative homo‐ and hetero‐γ‐coupling of α,β‐unsaturated aldehydes **54** and **54′**.

A stereoselective oxidative homo‐ and hetero‐γ‐coupling of in situ generated dienamines mediated by a catalytic amount of Cu(II) with atmospheric oxygen serving as a terminal oxidant was developed as outlined in Scheme 23 [106].

The protocol was amenable to α,β‐unsaturated aldehydes **54** and **54′** carrying both cyclic and acyclic aliphatic γ‐substituents and aromatic substituents on the β‐position to afford the coupled adducts **70** in good to high yield and stereoselectivity (Scheme 23). The proposed catalytic cycle is initiated by the condensation of *ent*‐**C11** onto **54** and **54′** affording dienamines **LIV** and **LIV′** which upon SET oxidation by Cu(II) generates radical cations **LXXIV** and **LXXIV′**. To complete the oxidative coupling, **LXXIV** couples with **LXXIV′** or **LIV′** to generate **70** after catalyst hydrolysis (for simplicity shown only for **LXXIV′**). To regenerate Cu(II), the Cu(I) formed in the SET step is reoxidized by atmospheric oxygen.

**SCHEME 24:**
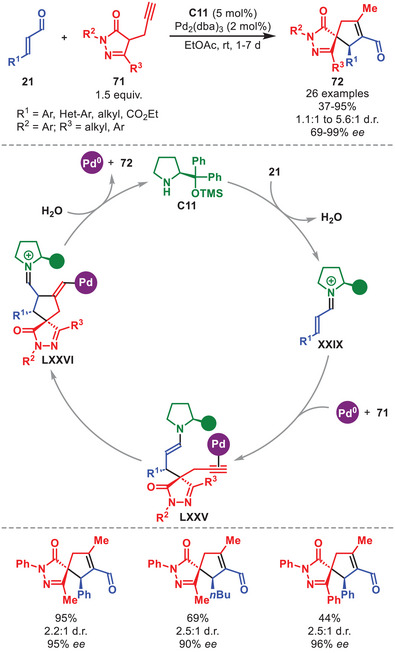
Pd‐catalyzed Michael/Conia‐ene cascade reaction between α,β‐unsaturated aldehydes **21** and pyrazolones **71**.

Synergistic amino‐ and palladium‐catalyzed systems have not only been employed in allylations, but also in for example, an asymmetric Michael/Conia‐ene cascade reaction (Scheme 24) [107]. In this work, Veselý et al. developed a methodology to access spirocyclic pyrazolones **72** in moderate to high yield and stereoselectivity from α,β‐unsaturated aldehydes **21**. The reaction was tolerant to both aromatic, aliphatic, and ester functionalities on **21**. For pyrazolones **71**, both aromatic substituent were suitable in R^2^ and R^3^, while aliphatic substituents were only viable for R^3^. The mechanism was suggested to proceed through an initial Michael addition onto the in situ generated iminium ion **XXIX** to afford intermediate **LXXV** upon complexation with Pd(0). The palladium mediated Conia‐ene reaction then proceeds to accomplish formation of **LXXVI**, which after hydrolysis and palladium release produces **72**, while regenerating the aminocatalyst.

**SCHEME 25:**
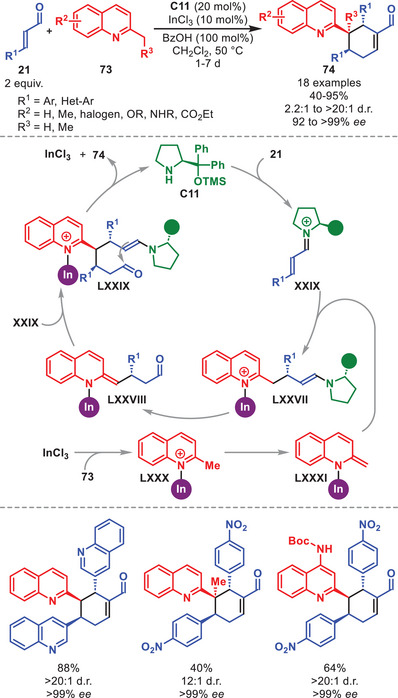
In‐catalyzed annulation between α,β‐unsaturated aldehydes **21** and alkyl quinolines **73**.

A synergistic strategy combining aminocatalytic iminium‐ion activation of aryl‐ and heteroaryl‐bearing α,β‐unsaturated aldehydes **21** with Lewis‐acid activation of substituted alkyl quinolines **73** was applied in 2017, enabling the access to both mono‐ and double‐addition adducts **74** in good to excellent yields and stereochemical outcomes (Scheme 25, only double addition shown) [108]. It was proposed that InCl_3_ first coordinates to the quinoline nitrogen to form **LXXX**, rendering the methyl proton more acidic, thereby facilitating the formation of intermediate **LXXXI**. Next, **LXXXI** attacks the in situ generated iminium ion **XXIX** to generate intermediate **LXXVII**. A second deprotonation leads to **LXXVIII** which can react with a second entity of **XXIX** to generate **LXXIX**. After intramolecular aldol condensation, hydrolysis of the aminocatalyst and decomplexation of the Lewis acid, **74** is formed and both catalytic entities are regenerated.

**SCHEME 26:**
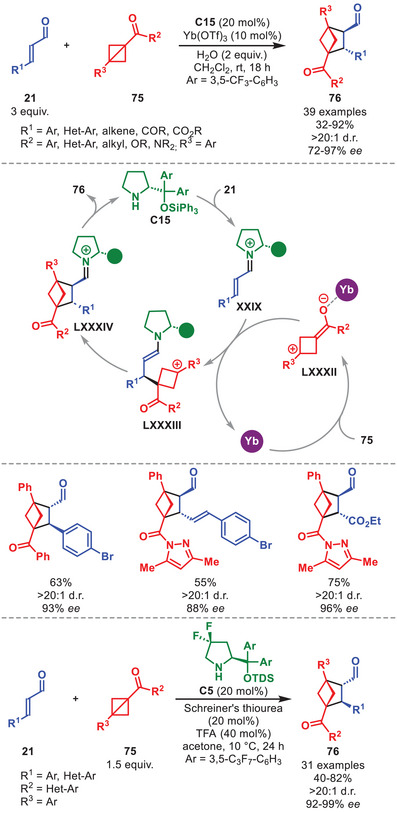
Top: Yb‐catalyzed [2+2] cycloadditions between α,β‐unsaturated aldehydes **21** and BCBs **75**. Bottom: Brønsted‐acid catalyzed variant.

Bicyclo[1.1.0]butanes (BCBs) are a class of strained rings that have garnered considerable attention in recent years [109, 110] due to their potential application for generating three‐dimensional scaffolds, valuable as bioisosteres within the “Escape from Flatland” paradigm [111, 112, 113]. The first usage of BCBs in combination with aminocatalysis was recently reported by our group utilizing a Lewis acid to synergistically activate BCBs **75** for a formal [2+2] cycloaddition with in situ generated iminium ions to generate highly functionalized bicyclo[2.1.1]hexanes (BCHs) **76** in moderate to high yield and enantioselectivity invariably as a single diastereoisomer (Scheme 26) [114].

The strategy could tolerate various α,β‐unsaturated aldehydes **21** bearing both aromatic and carbonyl substituents, but not aliphatic ones, and BCBs **75** carrying a variety of R^2^‐substituents, however electron‐deficient R^3^‐substituents were not tolerated (Scheme 26). After condensation of **C15** onto **21** to generate iminium ion **XXIX**, it was proposed that Yb(III) activates **75** to generate a zwitterionic intermediate **LXXXII** that can participate in a stereoselective nucleophilic attack on **XXIX** to afford **LXXXIII**. After ring‐closure and hydrolysis of the aminocatalyst, BCH **76** is released and the catalytic cycle can repeat.

Shortly after, it was shown by Xu et al. that a Brønsted acid and thiourea could likewise activate BCB **75** in a synergistic manner, enabling similar reactivity to afford BCHs **76** in good to high yield and excellent selectivity (Scheme 26, bottom) [115]. While the scope is narrower, the same limitation toward the substituent of α,β‐unsaturated aldehydes **21** and BCB **75** was observed. It is worth noting that it was found that the more sterically encumbered catalyst **C5** could furnish an even higher degree of enantioinduction.

**SCHEME 27:**
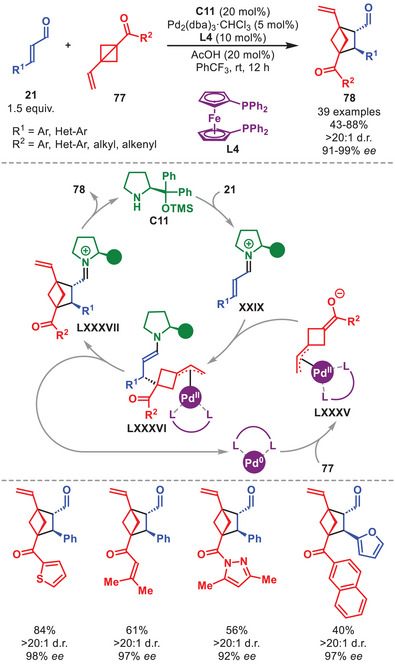
Pd‐catalyzed [2+2] cycloadditions between α,β‐unsaturated aldehydes **21** and BCBs **77**.

Additionally, it was showcased by the group of Feng et al. that vinyl‐BCBs **77** could be activated by palladium, enabling the formal [2+2] cycloaddition with aminocatalytically generated iminium ions to afford BCHs **78** in good to high yield and excellent stereocontrol (Scheme 27) [116].

The reaction tolerated aromatic α,β‐unsaturated aldehydes **21** but did not allow aliphatic substituents (Scheme 27). The vinyl‐BCBs **77** were amenable to ketone functionalities, however, did not allow esters, sulfones or the installation of bulk on the vinyl group. The mechanism is reminiscent of the previous example, the difference lying in the activation of **77**. It was proposed that **77** undergoes oxidative addition with Pd(0) generating the zwitterionic π‐allyl‐palladium intermediate **LXXXV**, which can then undergo 1,4‐addition to iminium ion **XXIX** to generate **LXXXVI**. Intramolecular allylic substitution generates intermediate **LXXXVII** while liberating Pd(0). Hydrolysis of **LXXXVII** liberates product **78** and aminocatalyst **C11**. It is worth noting that synergistic aminocatalytic and palladium mediated systems exploiting vinyl‐substituted strained rings have previously been reported for vinyl‐cyclopropanes [117, 118, 119] and vinyl‐aziridines [120].

**SCHEME 28:**
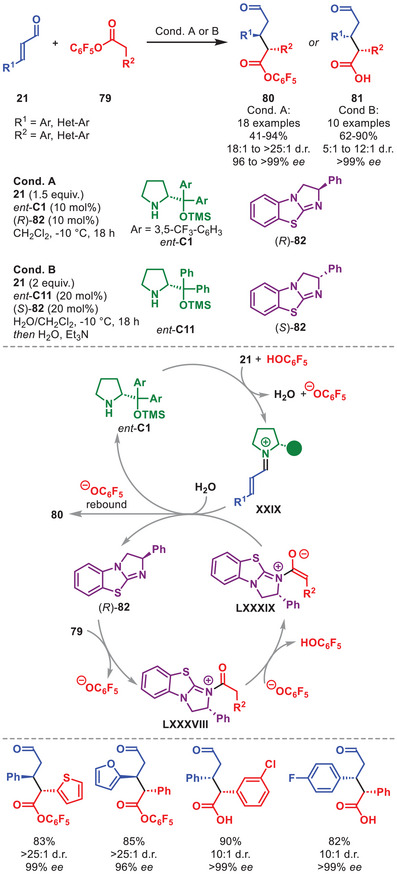
Benzotetramisole‐catalyzed stereodivergent Michael addition of aryl acetic acid esters **79** to α,β‐unsaturated aldehydes **21**.

Recently, a stereodivergent protocol exploiting a synergistic amino‐ and Lewis base–catalyst system was reported by the group of Lee for the Michael addition of aryl acetic acid esters **79** to aromatic α,β‐unsaturated aldehydes **21**, selectively enabling access to the *anti*‐ and *syn*‐configured adducts, **80** and **81**, respectively, in good to high yield and stereochemical outcomes (Scheme 28) [121]. While both protocols offered access to either product isomers in high stereochemical fidelity, generally the diastereoselectivity was higher for **80** (*anti*) compared to the *syn*‐isomer. The proposed catalytic cycle starts with a condensation of *ent*‐**C1** to **21**, providing iminium ion **XXIX**. Simultaneously, benzotetramisole (*R*)‐**82** undergoes acyl substitution to the aryl acetic acid ester **79** to generate intermediate **LXXXVIII**. Upon deprotonation of **LXXXVIII**, the enolate **LXXXIX** is unveiled and undergoes nucleophilic attack to **XXIX**. Upon hydrolysis of the aminocatalyst, and acyl substitution by the aryloxide, *anti*‐adduct **80** is released and both organocatalysts regenerated. It was also shown that the adducts could be further functionalized by an aminocatalytic α‐fluorination, either after isolation or one‐pot, forging a total of three contiguous stereocenters with excellent stereoselectivity.

The same authors also accomplished a stereodivergent Michael addition between α,β‐unsaturated aldehydes and α‐fluoro azaaryl acetamides relying on a chiral Lewis acid complex to activate and direct the enolate precursor in combination with iminium‐ion activation, accessing all four possible stereoisomers with high chemical fidelity [122].

**SCHEME 29:**
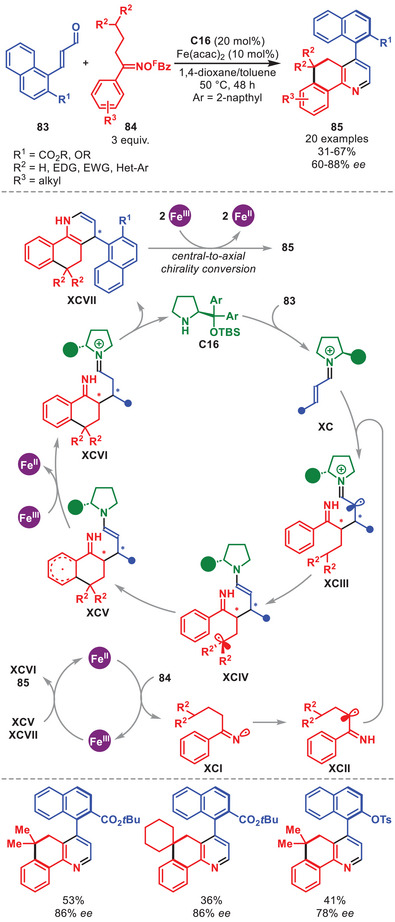
Fe‐catalyzed atroposelective annulation between α,β‐unsaturated aldehydes **83** and oxime esters **84**.

A synergistic concept exploiting the 1,5‐HAT of a formed α‐iminyl radical to enable the generation of enantioenriched axially chiral heterobiaryls **85** in moderate to good yields and enantioselectivitity was described (Scheme 29) [123]. The reaction was built on the non‐enantioselective protocol for cinnamaldehyde derivatives reported in the same paper. Hence, the authors utilized napthyl‐derived α,β‐unsaturated aldehydes **83** embedding an ester or ether *ortho*‐substituent with oxime esters **84** amenable for both electron‐rich and ‐poor aryl substituents. In the proposed mechanism **C16** first condenses to **83** to generate iminium ion **XC**. Simultaneously, a SET reduction of **84** proceeds to afford iminyl radical **XCI**, while oxidizing Fe(II) to Fe(III). Next, **XCI** undergoes 1,3‐HAT to form α‐carbon radical **XCII** which engages in conjugate addition with **XC** affording **XCIII**. Subsequent 1,5‐HAT generates the tertiary radical **XCIV**, followed by homolytic aromatic substitution providing **XCV**. SET oxidation by Fe(III) and tautomerization leads to **XCVI**, which by an intramolecular cyclization and aminocatalyst elimination generates centrally chiral intermediate **XCVII** and regenerates the aminocatalyst. Upon two sequential single‐electron oxidations, atropoisomer **85** is generated through a central‐to‐axial chirality conversion.

**SCHEME 30:**
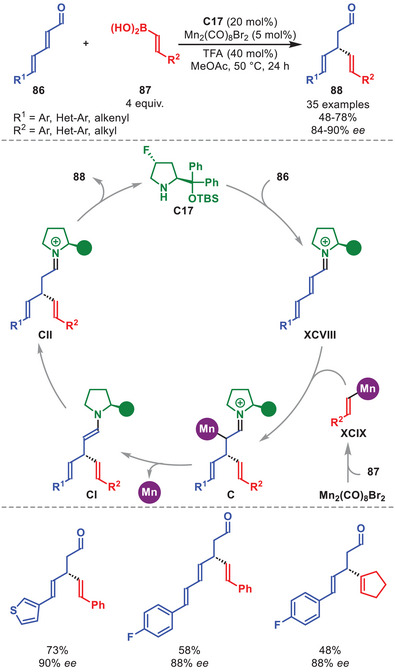
Mn‐catalyzed β‐alkenylation of dienals **86** with alkenyl boronic acids **87**.

In 2024, the synergistic Mn(I)‐ and aminocatalytic enantioselective C(*sp^2^*)─C(*sp^3^*) bond formation to generate skipped dienes **88** from dienals **86** and alkenyl boronic acids **87** in good to high yields and high enantioselectivity was presented (Scheme 30) [124]. The methodology was tolerant for **86** carrying aromatic or styrenyl residues, while **87** can accommodate both aromatic and aliphatic substituents. The proposed mechanistic cycle is initiated by the condensation of **C17** to **86** to generate vinylogous iminium ion **XCVIII**. Simultaneously, the dimeric manganese source undergoes transmetallation with **87** to generate a HOMO‐raised alkenylmanganese species **XCIX**. Migratory insertion into the C─[Mn] bond of **XCIX** leads to intermediate **C**, which after demetallation and tautomerization forms iminium ion **CII**. Hydrolysis regenerates the aminocatalyst and liberates the skipped diene **88**. Intriguingly, the propensity for the 1,4‐ over 1,6‐hydroalkenylation was investigated by DFT calculations indicating that while both pathways are thermodynamically feasible, a kinetic bias toward the 1,4‐addition is present.

**SCHEME 31:**
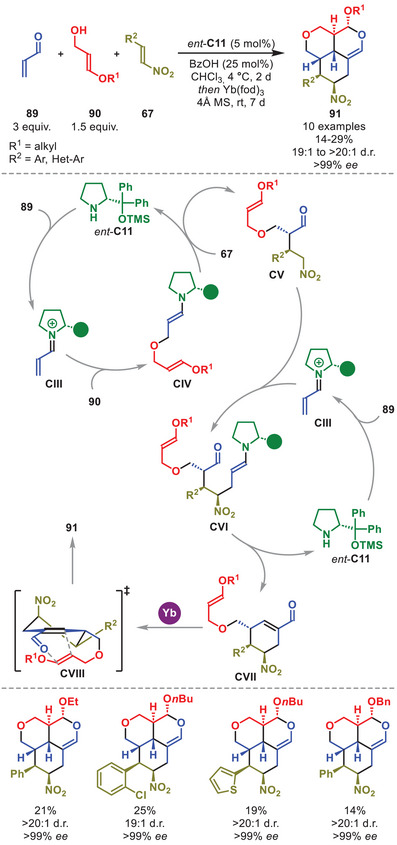
Yb‐catalyzed, multi‐component cascade annulation of acrolein **89**, alcohols **90** and nitrostyrenes **67**.

Although synergistic catalytic protocols are the most common, examples have been reported involving sequential procedures to generate complex product scaffolds. Enders et al. presented a quadruple component cascade followed by a sequential Lewis acid‐mediated hetero‐inverse electron‐demand Diels–Alder reaction (IEDDA) to be feasible in a one‐pot procedure (Scheme 31) [125].

The transformation is commenced by the condensation of *ent*‐**C11** to acrolein **89** to generate iminium ion **CIII**, which is susceptible to an *oxa*‐Michael addition from alcohol **90** to generate enamine intermediate **CIV** (Scheme 31). Upon Michael addition of enamine **CIV** to nitroolefin **67** followed by hydrolysis of the aminocatalyst, the nucleophilic intermediate **CV** is formed. Upon Michael addition of **CV** to another equivalent of **CIII**, intermediate **CVI** is generated, which upon intramolecular aldol condensation forms the isolable intermediate **CVII** after hydrolysis of the aminocatalyst. Upon subjection of **CVII** to Yb(III), either one‐pot or after isolation, a Lewis acid‐mediated stereospecific hetero‐IEDDA reaction (**CVIII**) proceeds to generate the complex tricyclic cycloadduct **91**. While the yields were modest, credit should be given, as the overall process is a highly elaborate and stereoselective multicomponent reaction from commercial or easily accessible substrates.

While marking perhaps the most prominent example of sequential catalysis based on aminocatalysis in the last decade, other examples involving Lewis acids [126, 127] and *N*‐heterocyclic carbene catalysis [128] have also been reported.

## 5 Higher‐Order Cycloadditions

Cycloadditions involving more than 6π‐electrons are termed higher‐order cycloadditions (HOCs). The feasibility of thermal HOCs was already postulated as early as the Woodward and Hoffmann papers detailing the selectivity rules of pericyclic reactions based on orbital symmetry considerations [129, 130]. One year after its prediction, the first [6+4] cycloaddition was experimentally accomplished independently by the groups of Cookson and Itô detailing the reaction between tropone and cyclopentadiene [131, 132]. Classically, HOCs were notorious for affording poor yields and periselectivities; however, since the increased application of catalytic systems, several highly selective methods have been developed [133, 134, 135, 136].

The first enantioselective intermolecular organocatalytic protocols were reported serendipitously in 2017 by our group and Chen et al., and since then the field has undergone a renaissance [137, 138]. In particular, the application of aminocatalysis in the development of new enantioselective HOCs has been exploited, often applying aminocatalytic species containing 10π‐electrons or more.

**SCHEME 32:**
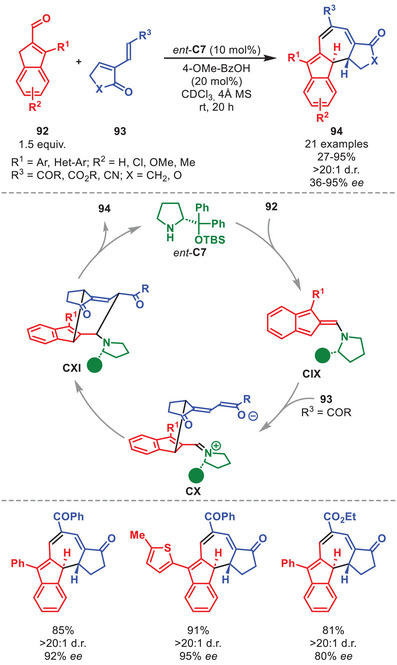
[10+4] Cycloaddition between indenecarbaldehydes **92** and dienes **93**.

One of the earliest publications regarding aminocatalytic HOCs utilizing the diarylprolinol silyl ether catalyst *ent*‐**C7** was disclosed in 2018, presenting the [10+4] cycloaddition between indenecarbaldehydes **92** and electron‐deficient dienes **93** (Scheme 32) [139]. This cycloaddition afforded dihydroazulenes **94** in moderate to excellent yields and enantioselectivity invariably as single diastereoisomers. Multiple substitution patterns on **92** were tolerated, while various electron‐withdrawing groups on **93** were amenable. DFT calculations showed that the stereoinduction originates from a kinetic preference toward cyclic polyenamine intermediate **CIX** over other competing conformation. This was further corroborated by the beneficial effect of removing moisture by adding molecular sieves as hydrolysis back to the substrates is minimized. Intermediate **CIX** can then undergo a step‐wise [10+4] cycloaddition to generate **CXI**, which, after elimination of the catalyst, releases **94**.

**SCHEME 33:**
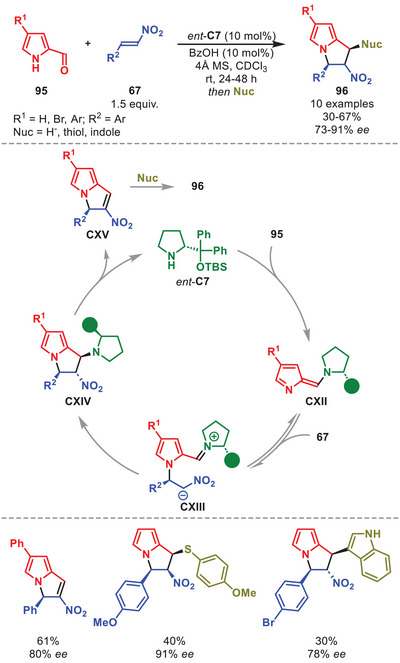
[6+2] Cycloaddition between pyrrole carbaldehydes **95** and nitrostyrenes **67**.

It was also demonstrated that aminocatalytically generated aza‐ and diazafulvenes could react as electron‐rich 6π‐components in stereoselective [6+2] and [6+4] cycloadditions of pyrrole carbaldehydes **95** with nitroolefins **67** and electron‐deficient dienes, respectively. However, only the [6+2] cycloaddition utilized a diarylprolinol silyl ether catalyst, therefore the [6+4] cycloaddition will not be discussed herein. The catalytic cycle is initiated by the aminocatalytic formation of azafulvene intermediate **CXII** from **95** and *ent*‐**C7** (Scheme 33) [140]. DFT calculations showed that **CXII** engages in a non‐selective addition to **67** to generate both enantiomers of intermediate **CXIII**. However, only the (*R*)‐configured intermediate **CXIII** can proceed as suggested by the calculated transition state barrier for the second‐bond formation being nearly 10 kcal mol^−1^ lower in energy than the (*S*)‐configured **CXIII**, therefore ensuring the selective generation of intermediate **CXIV**. Upon catalyst elimination, **CXV** is generated, which upon treatment with a nucleophile can provide **96**. The cycloadducts **96** were formed in moderate to good yields and high enantioselectivity. The [6+2] cycloaddition was amenable to pyrrole carbaldehydes carrying various substitution patterns. Multiple nucleophiles can be employed such as hydride, thiols, and indole; however, treatment with a nucleophile was not required and can be stopped by isolation of **CXV**.

**SCHEME 34:**
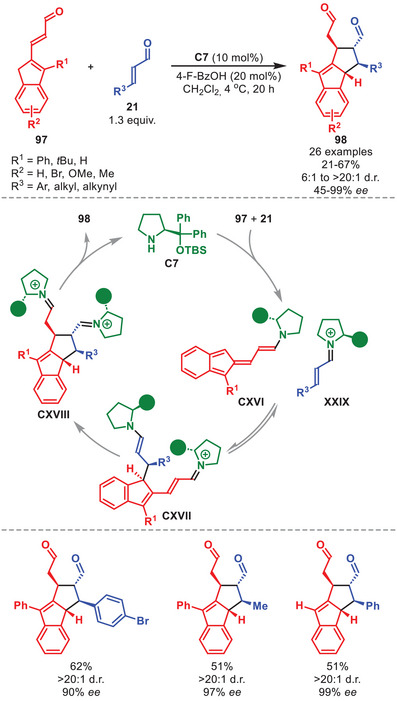
[10+2] Cycloaddition between homologated indenecarbaldehydes **97** and α,β‐unsaturated aldehydes **21**.

In 2020, the stereoselective [10+2] cycloaddition to afford chiral tetrahydrocyclopenta[*a*]indenes **98** in low to good yields and moderate to excellent stereocontrol was achieved by applying catalyst **C7** (Scheme 34) [141]. This was accomplished in a dual catalytic fashion where **C7** simultaneously activates both the α,β‐unsaturated aldehyde **21** and homologated indenecarbaldehyde **97**, as revealed by the presence of a significant non‐linear effect. Indenecarbaldehydes **97** bearing a variety of substitution patterns could be employed, as did **21** carrying both aliphatic‐, aromatic‐, and unsaturated substituents. Upon aminocatalytic activation of **97** and **21** to generate **CXVI** and **XXIX**, the step‐wise [10+2] cycloaddition proceeds furnishing **CXVIII**, which releases **98** upon hydrolysis of the catalysts. The step‐wise mechanism was further corroborated by the isolation of an intermediate after the first bond formation (intermediate **CXVII** after hydrolysis of the aminocatalysts). It was also found through DFT calculations, that the cycloaddition operates through a Curtin–Hammett scenario where the possible stereoisomers of **CXVII** are in equilibrium enabling only the isomer capable of inducing the final stereochemistry to proceed through the ring‐closure generating **CXVIII**.

**SCHEME 35:**
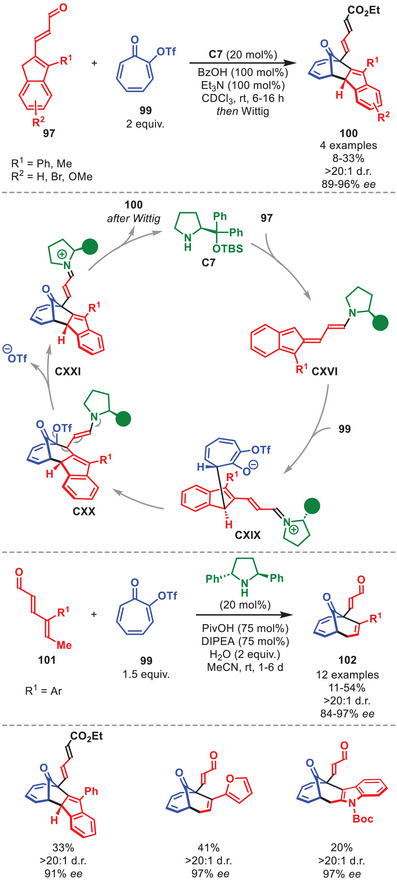
Top: [10+6] Cycloaddition between homologated indenecarbaldehydes **97** and 2‐trifluoromethanesulfonate tropone **99**. Bottom: [6+4] Cycloaddition between dienals **101** and **99**.

The diarylprolinol silyl ether was shown to control the HOCs between 2‐trifluoromethanesulfonate tropone **99** and various vinylogous enamine species (Scheme 35) [142]. It was demonstrated as a proof‐of‐concept that **99** could undergo an unprecedented [10+6] HOC with homologated indenecarbaldehydes **97** in the presence of **C7**, followed by an unprecedented ring‐contracting Favorskii‐like rearrangement to afford cycloadducts **100** with high peri‐ and stereoselectivity. Although, the reaction was accomplished in low yield, it allowed for the formation of complex cycloadducts with high stereoselectivity. The mechanism of the transformation was investigated in detail by DFT calculations but will not be discussed in detail herein.

The catalytic cycle in Scheme 35 begins with the condensation of catalyst **C7** to the homologated indenecarbaldehydes **97** to generate vinylogous enamine **CXVI**, which then undergoes a step‐wise [10+6] cycloaddition with 2‐trifluoromethanesulfonate tropone **99** to generate transient cycloadduct **CXX**. Upon rearrangement and hydrolysis of **C7** cycloadduct **100** is formed. Furthermore, it was shown that **99** could undergo a similar cascade reaction with dienals **101** operating through a trienamine catalyzed [6+4] cycloaddition followed by an equivalent Favorskii‐like rearrangement to generate **102** in high peri‐ and stereoselectivity and moderate yields. Only aromatic substituents on the dienal **101** could facilitate the rearrangement (Scheme 35, bottom).

**SCHEME 36:**
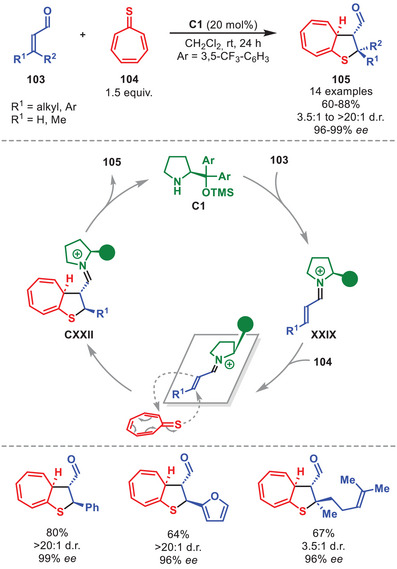
[8+2] Cycloaddition between α,β‐unsaturated aldehydes **103** and tropothione **104**.

In 2019, the group of Albrecht disclosed an aminocatalytic [8+2] cycloaddition between tropothione **104** and α,β‐unsaturated aldehydes **103**, having one or more aliphatic and aromatic β‐substituents, enabled by iminium‐ion catalysis to stereoselectively generate chiral tetrahydrothiophenes **105** in good to high yield and generally excellent stereocontrol (Scheme 36) [143]. Pivotal for the success of this strategy was the incorporation of a sulfur atom into the tropone core, rendering the otherwise electron‐deficient 8π‐component instead electron‐rich. In the proposed mechanism, **C1** first condenses to **103** to generate iminium ion **XXIX**. Subsequently, the stereoselective [8+2] cycloaddition proceeds generating **CXXII**, which after catalyst hydrolysis liberates **105**. The diastereocontrol arises from the desymetrization of **104** via an approach between the prochiral faces of **104** and **XXIX** during the cycloaddition to minimize steric interactions with the exocyclic group of **C1**.

**SCHEME 37:**
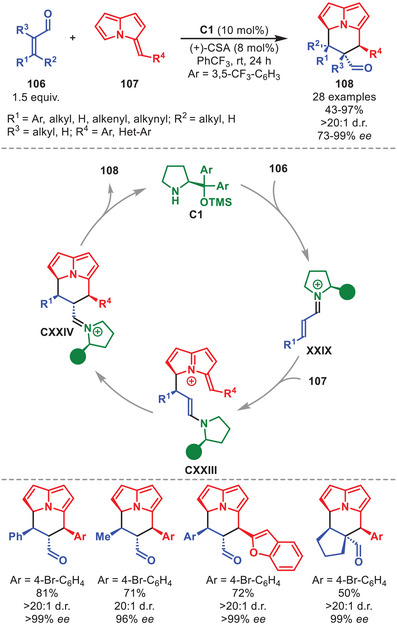
[8+2] Cycloaddition between α,β‐unsaturated aldehydes **106** and benzylidene pyrrolizines **107**.

In 2021, an efficient synthesis of chiral cycl[3.2.2]azines **108** in a highly stereoselective manner in moderate to high yields based on the use of diarylprolinol silyl ether was disclosed (Scheme 37) [144]. This was accomplished by the utilization of (*E*)‐3‐benzylidene‐3*H*‐pyrrolizines **107**, embedding aromatic substituents, in conjunction with α,β‐unsaturated aldehydes **106** catalyzed by **C1** through an iminium‐ion mediated step‐wise [8+2] cycloaddition. The α,β‐unsaturated aldehydes were tolerant toward a variety of substitution patterns, including aliphatic and aromatic groups. After condensation of **C1** to **106**, the iminium ion **XXIX** undergoes a step‐wise [8+2] cycloaddition with **107** affording intermediate **CXXIV**, which after hydrolysis liberates cycl[3.2.2]azine **108** and regenerates the catalyst.

## 6 Total Synthesis

Within the last decade aminocatalytic strategies based on diarylprolinol silyl ethers as catalysts have been exploited on multiple occasions for the total syntheses of complex natural compounds [30]. As this review will not contain a comprehensive examination of all total syntheses utilizing this class of catalysts in one or more of its synthetic steps, some selected examples involving a key aminocatalytic step will be discussed.

**SCHEME 38:**
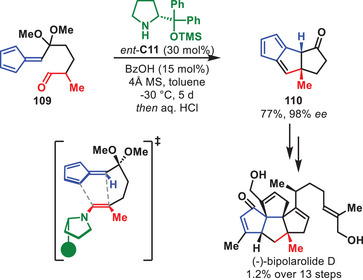
Intramolecular [6+2] cycloaddition in the total synthesis of (‐)‐bipolarolide D.

One impressive accomplishments of aminocatalysis in total synthesis over the last decade was disclosed by the group of Lu in 2024 detailing the synthesis of (‐)‐bipolarolide D (Scheme 38) [145]. In this synthesis, the key step involved an aminocatalytic intramolecular [6+2] HOC between an enamine and pentafulvene motif, inspired by the first reported intramolecular aminocatalytic HOC showcased by Hayashi et al. in 2011 [146]. By slight modification of Hayashi's original conditions, the authors could successfully access **110** in 77% yield and 98% *ee* from **109** in a one‐pot [6+2] HOC and ketal hydrolysis procedure. Ultimately, this allowed for the first enantioselective synthesis of (‐)‐bipolarolide D in 1.2% yield over 13 steps.

**SCHEME 39:**
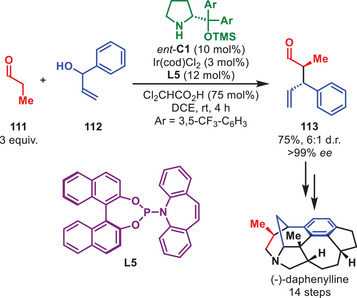
Ir‐catalyzed α‐allylation in the total synthesis of (‐)‐daphenylline.

Dual catalytic α‐allylations have been utilized as a key step to introduce chiral information at an early stage in multiple total syntheses over the past decade [147, 148]. A recent example was presented by the group of Yang for the total synthesis of (‐)‐daphenylline (Scheme 39) [149]. Their total synthesis was initiated by the formation of chiral aldehyde **113** from propanal **111** and phenyl vinyl carbinol **112** following a modification of the Ir/aminocatalyst synergistic system developed by Carreira [86, 87, 88]. Aldehyde **113** was accessed in 75% yield, 6:1 d.r. and >99% *ee* after optimization of the acid additive. Access to this key intermediate enabled the enantioselective total synthesis of (‐)‐daphenylline in 14 steps.

**SCHEME 40:**
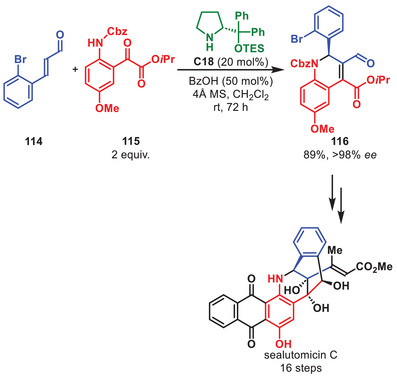
*Aza*‐Michael/aldol cascade in the total synthesis of sealutomicin C.

Recently, the group of Burton developed an aminocatalytic enantioselective *aza*‐Michael/aldol cascade for the synthesis of dihydroquinolines **116** in 89% yield and greater than 98% *ee* (Scheme 40). The dihydroquinoline synthesis proved compatible with other substitution patterns of aromatic α‐ketoesters **115** and cinnamaldehyde derivatives **114** with high yields and excellent selectivity throughout the presented scope. This subsequently allowed for the total synthesis of sealutomicin C in 16 steps as the longest linear sequence [150]. An intramolecular cyclization of an aryllithium onto a γ‐lactone is featured as a second key step.

**SCHEME 41:**
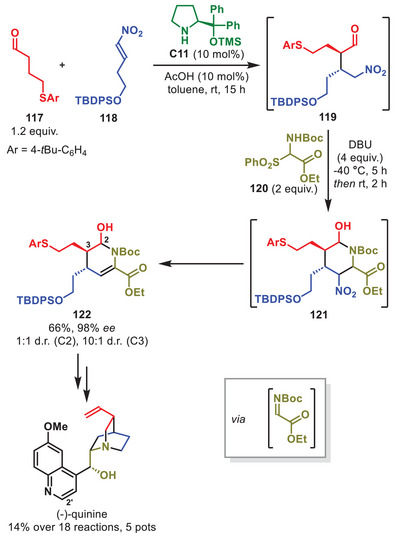
Multicomponent Michael/*aza*‐Henry cascade in the total synthesis of (‐)‐quinine.

Recently, Hayashi et al. accomplished a highly pot‐efficient total synthesis of (‐)‐quinine in 14% total yield utilizing a key multicomponent aminocatalytic step to build part of the core of the quinuclidine motif (Scheme 41) [151]. The first of five one‐pot procedures involves a three component Michael/*aza*‐Henry cascade catalyzed by **C11**. The multicomponent cascade reaction was initiated by the aminocatalytic Michael addition between the enamine generated from aldehyde **117** and nitroolefin **118** to form intermediate **119**. Upon addition of imine precursor **120** and DBU to the same pot, the *aza*‐Henry/hemiaminalization cascade proceeded to form piperidine **121**, which after elimination of the nitro group by DBU, generated tetrahydropyridine **122** in 66% yield and excellent stereoselectivity as the C2‐isomer is inconsequential since the hydroxy group is subsequently reductively removed. As the quinoline motif was installed late, Hayashi et al. also showed that installation of a bromine in the C2′‐position enabled access to multiple (‐)‐quinine derivatives.

**SCHEME 42:**
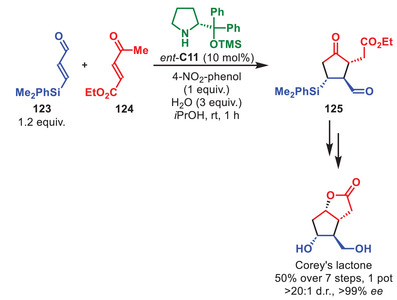
Michael/Michael cascade in the total synthesis of Corey's lactone.

In 2020, the group of Hayashi accomplished the seven step, one‐pot synthesis of Corey's lactone leveraging an aminocatalytic Michael/Michael cascade to introduce the stereochemical information (Scheme 42) [152]. This was achieved using acrylate **124** and α,β‐unsaturated aldehyde **123** to generate cyclopentanone **125** in the presence of *ent*‐**C11** in the first step of the multi‐step one‐pot protocol. This enabled the synthesis of Corey's lactone in 50% overall yield as a single stereoisomer. Furthermore, the authors demonstrated that the Michael/Michael cascade to generate cyclopentanone motifs was also feasible for different α,β‐unsaturated aldehydes, including cinnamaldehyde derivatives.

**SCHEME 43:**
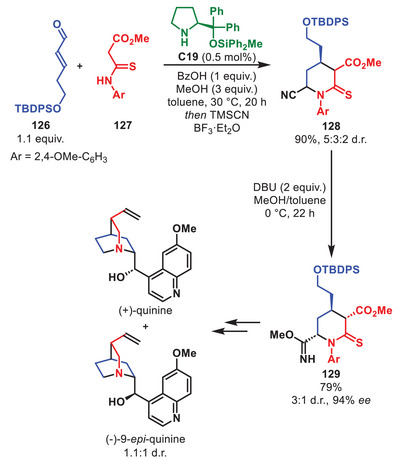
*Aza*‐[3+3] annulation in the total synthesis of (+)‐quinine and (‐)‐9‐*epi*‐quinine.

The total synthesis of the unnatural isomer of quinine, (+)‐quinine, as well as (‐)‐9‐*epi*‐quinine, was accomplished by the group of Ishikawa (Scheme 43) [153]. Cornerstone to this was a formal *aza*‐[3+3] annulation catalyzed by only 0.5 mol% of **C19** to trigger the annulation between α,β‐unsaturated aldehyde **126** and thiomalonamate **127**. The resulting cyano Δ‐thiolactam **128** was afforded in high yield as a mixture of three diastereoisomers; however, treatment with MeOH and DBU afforded imidate **129** in 79% yield, 3:1 d.r. and 94% *ee* of each diastereoisomer. This ultimately enabled the synthesis of both (+)‐quinine and (‐)‐9‐*epi*‐quinine in 16% overall yield as a 1.1:1 mixture, although importantly, it was demonstrated that the two quinine isomers could efficiently be interconverted by a Mitsunobu reaction.

**SCHEME 44:**
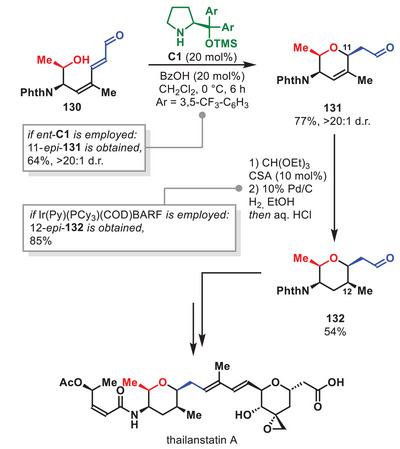
Intramolecular *oxa*‐Michael in the total synthesis of thailanstatin A.

Nicolaou et al. utilized a biomimetic intramolecular aminocatalytic *oxa*‐Michael reaction to diastereodivergently access highly functionalized dihydropyrans, which could further be hydrogenated to tetrahydropyrans, thereby proving pivotal in the total synthesis of thailanstatin A (Scheme 44) [154]. When dienal **130** was subjected to **C1** dihydropyran **131** was accessed as a single diastereoisomer in 77% yield. Should *ent*‐**C1** instead be utilized the C11‐epimer 11‐*epi*‐**131** was afforded as a single diastereoisomer in 64% yield. Hydrogenation of dihydropyran **131** by Pd/C under H_2_ atmosphere generated tetrahydropyran **132** in 54% over three steps. Replacing Pd/C with Ir(Py)(PCy_3_)(COD)BARF, the C12‐epimer 12‐*epi*‐**132** was accessed in 85% overall yield. Finally, thailanstatin A was obtained by a nine step longest linear sequence. Later, the group of Nicolaou demonstrated that this strategy could be applied to generate multiple analogous and assessed their antitumor potency against multiple cell lines [155].

**SCHEME 45:**
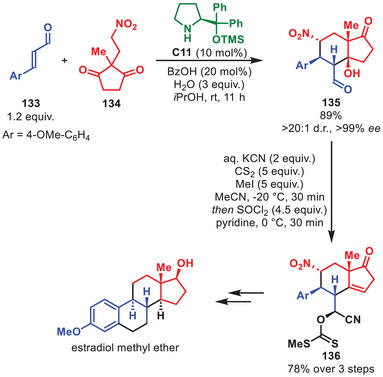
Michael/aldol cascade in the total synthesis of estradiol methyl ether.

The group of Hayashi applied **C11** to introduce stereochemical information in the pot‐efficient total synthesis of estradiol methyl ether in 15% overall yield over 15 steps in only five reaction vessels (Scheme 45) [156]. The total synthesis was initiated by the aminocatalytic Michael/aldol cascade between α,β‐unsaturated aldehyde **133** and nitroalkane **134** to generate bicyclo[4.3.0]nonane **135** in 89%, >20:1 d.r. and >99% *ee*. It should be noted that this intermediate need not be isolated in the total synthesis of estradiol methyl ether and could be carried through a three step, one‐pot procedure to afford intermediate **136** in 78% yield. Furthermore, it was also demonstrated that the initial Michael/aldol cascade was general and could amend different cinnamaldehydes in high yield and with excellent stereochemical outcome.

## 7 Miscellaneous

Beyond the novel activation modes unlocked through the combination of diarylprolinol silyl ether catalysts with emerging technologies such as photo‐ and electrochemistry, their synergy with metal catalysis, their application in higher‐order cycloadditions, and their practical utility in enabling key steps of total syntheses, recent years have also experienced the continued application of classic aminocatalytic activation manifolds. Traditional activation modes, such as enamine, dienamine, trienamine, and iminium‐ion catalysis have remained fundamental for developing new enantioselective transformations—particularly within aminocatalyzed cycloaddition reactions—providing access to novel, chiral, cyclic chemotypes, as well as bio‐relevant polycyclic scaffolds [29]. These advances underscore how the diarylprolinol silyl ether family persists as an evergreen and powerful platform for expanding accessible three‐dimensional chemical space. In this chapter, we will highlight some recent and relevant contributions, providing an overview of how classical organocatalytic activation modes have enabled novel enantioselective cycloaddition or annulation reactivity, through unprecedented mechanistic pathways.

**SCHEME 46:**
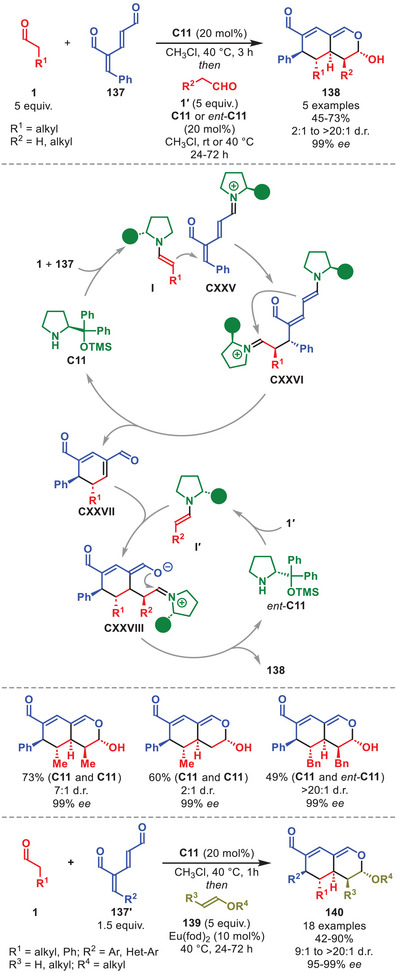
Top: Multicomponent, cascade between aldehydes **1**, **1′** and dienal **137**. Bottom: Eu‐catalyzed variant using vinyl ethers **139**. fod = 6,6,7,7,8,8,8‐heptafluoro‐2,2‐dimethyl‐3,5‐octanedionate.

In 2016, a three‐component, one‐pot cascade process combining enamine and vinylogous iminium‐ion catalysis to enable a sequential IEDDA reaction between aldehydes **1**, **1′** and oxadendralenes **137**, catalyzed by **C11** was unveiled (Scheme 46) [126]. This strategy furnished a series of optically active tetrahydroisochromenes **138** in moderate to good yields and invariably with full enantioselectivity, with the diastereoselectivity depending on whether aldehyde **1** was prochiral, and on the use of **C11** *vs. ent*‐**C11** in the second step.

The transformation relies on dual activation of both reacting partners by two molecules of the same catalyst (Scheme 46). Activation of oxadendralene **137** by **C11** generates a vinylogous iminium ion **CXXV**, which undergoes a regioselective 1,6‐addition with enamine **I**, formed from aldehyde **1** and a second molecule of **C11**. The resulting intermediate **CXXVI** engages in an intramolecular cyclization to form cyclic oxadendralenic intermediate **CXXVII**, which acts as the dienophile in a subsequent enamine‐mediated hetero‐IEDDA reaction. Use of *ent‐* **C11** in this second cycloaddition leads to an *exo*‐transition state *via* (*E*‐s‐*trans*) conformation of sterically demanding aldehydes, thereby enhancing diastereoselectivity. Within the same study, it was also demonstrated that intermediate **CXXVII** could be used in combination with Eu(fod)_2_ as a Lewis acid to promote a hetero‐IEDDA reaction with vinyl ethers **139**, affording tetrahydroisochromenes **140** bearing five contiguous stereocenters with excellent enantioselectivity.

**SCHEME 47:**
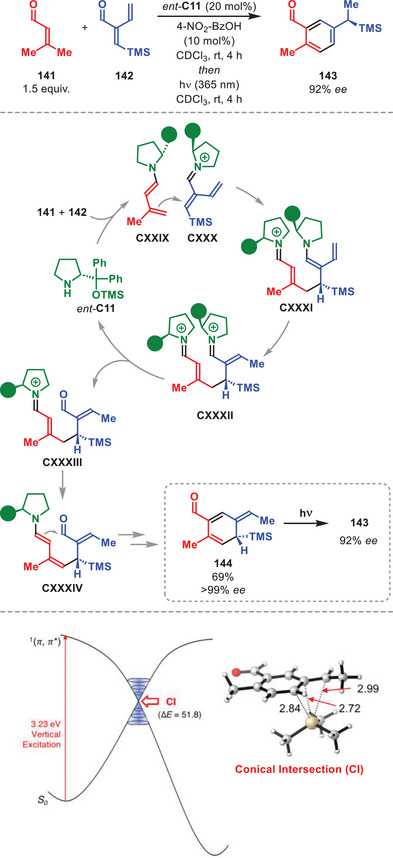
Top: Photochemical [1,3]‐sigmatropic silyl shift of allylsilane **144**. Bottom: Silyl/allyl conical intersection of **144**.

A distinctive example at the interface of organic methodological and computational chemistry involving diarylprolinol silyl ether catalysis was presented in 2020 (Scheme 47). Here, the first stereoselective photochemical [1,3]‐sigmatropic silyl shift of an allylsilane was disclosed [157]. In this investigation an organocatalytic enantioselective cascade annulation between 3‐methylcrotonaldehyde **141** and oxadendralene **142**, catalyzed by *ent‐* **C11**, enabled the formation of a chiral enantiopure silyl‐*o*‐isotoluene **144** (Scheme 47). The reaction proceeds through a dual aminocatalytic activation, involving formation of a dienamine **CXXIX** and a rare cross‐iminium‐ion **CXXX**, followed by a stereoselective conjugate addition, intramolecular aldol cyclization, and dehydration to furnish *Z*‐s‐*cis*‐configured silyl‐*o*‐isotoluene **144**. Subsequently, UV irradiation promoted an unprecedented photochemical [1,3]‐silyl shift, delivering enantioenriched benzylsilane **143** with only minimal erosion of stereochemical integrity. CASSCF, DFT, and TD‐DFT computational studies revealed that the photochemical rearrangement proceeds via photoexcitation to a singlet excited state, followed by ultrafast internal conversion through a silyl/allyl conical intersection (CI). The excited‐state surface becomes degenerate with the ground‐state surface in a product‐like geometry, directing the system toward formation of the [1,3]‐silyl‐shifted benzylsilane **143**. Moreover, control experiments employing radical traps, triplet sensitizers, and triplet quenchers were consistent with a non‐radical, singlet excited‐state pathway governed by a conical intersection.

**SCHEME 48:**
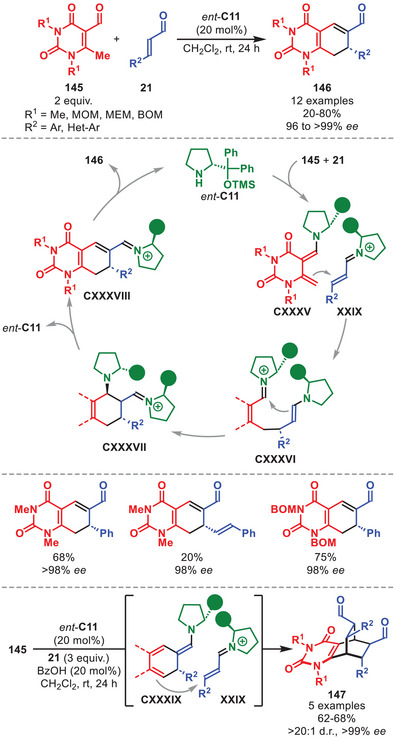
Top: [4+2] cycloaddition between 6‐methyluracil‐5‐carbaldehydes **145** and α,β‐unsaturated aldehydes **21**. Bottom: Double [4+2] cycloaddition variant.

In 2020, Zanardi et al. reported an aminocatalytic cross‐[4+2] cycloaddition between 6‐methyluracil‐5‐carbaldehydes **145** and α,β‐unsaturated aldehydes **21**, enabling the enantioselective synthesis of dihydroquinazoline‐2,4‐diones **146** catalyzed by *ent*‐**C11** (Scheme 48) [158]. A series of cycloadducts **146** were obtained in moderate to high yields with high enantioselectivity. Substituent variation at the N1‐ and N3‐positions of **145** was well tolerated.

Mechanistic investigations and DFT calculations revealed a dual catalytic activation mode in which both reaction partners 6‐methyluracil‐5‐carbaldehydes **145** and α,β‐unsaturated aldehydes **21** are simultaneously activated by two molecules of *ent‐* **C11**, forming the corresponding uracil‐based dienamine *ortho‐*quinodimethane **CXXXV** and iminium ion **XXIX**, respectively (Scheme 48). Dihydroquinazoline‐2,4‐dione **146** arose from a step‐wise, eliminative [4+2] cycloaddition initiated by a vinylogous Michael addition between dienamine **CXXXV** and iminium ion **XXIX**, generating **CXXXVI**. This intermediate undergoes an intramolecular cyclization to furnish bicyclic species **CXXXVII**, which readily evolves into the iminium ion **CXXXVIII** via elimination of one catalyst molecule. Subsequent hydrolysis releases the second equivalent of *ent‐* **C11** to furnish **146**. The methodology was further extended to the synthesis of uracil‐fused bicyclo[2.2.2]octanes **147**. When the reaction was performed using an excess of **21** in the presence of benzoic acid, a double [4+2] cycloaddition took place, affording enantiopure **147**. Under these conditions, isomerization of intermediate **CXXXVIII** into the corresponding trienamine *ortho‐*quinodimethane **CXXXIX** is favored, enabling a subsequent cycloaddition with a second molecule of chiral iminium ion **XXIX**, thereby leading to the tricyclic framework within **147** (Scheme 48, bottom).

**SCHEME 49:**
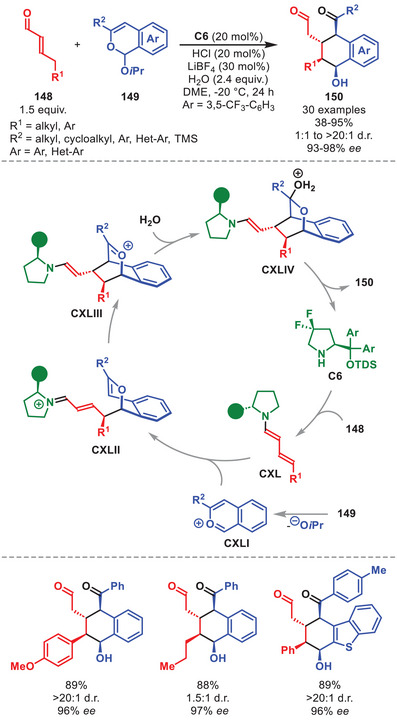
Multicomponent [4+2] cycloaddition between α,β‐unsaturated aldehydes **148** and acetals **149**.

Beyond this example, the use of diarylprolinol silyl ether catalysts has recently enabled the stereoselective synthesis of further novel polycyclic chemotypes exploiting heterocyclic *ortho‐*quinodimethane intermediates in [4+2]‐cycloadditions [159, 160, 161].

Recently, we reported an organocatalytic asymmetric multicomponent cascade based on dienamine catalysis, integrating a [4+2] cycloaddition, a nucleophilic addition, and a ring‐opening event into a single transformation. The unprecedented application of isobenzopyrylium ions **CXLI** in asymmetric catalysis, in combination with α,β‐unsaturated aldehydes **148** and catalyst **C6** in the presence of excess water, enabled access to chiral tetrahydronaphthols **150** bearing four contiguous stereocenters (Scheme 49) [162]. The cycloadducts **150** were obtained in good to high yields, with high diastereoselectivity, and excellent enantioselectivity. The reaction exhibited a broad substrate scope, tolerating a range of β‐alkyl‐ and β‐aryl‐substituted **148**, as well as aryl‐ and heteroaryl‐substituted acetals **149**. Interestingly, selected compounds were evaluated in U‐2OS cancer cells, where they induced clear morphological changes. Mechanistic insights were obtained through a combination of oxygen‐18‐labeling studies and DFT calculations. These studies supported a scenario in which intermediate **CXLI** is generated via expulsion of the isopropoxide leaving group from **149**. The electrophilic species **CXLI** is then intercepted at the γ‐position of dienamine **CXL**, formed from **C6** and **148**, forging the initial C─C bond and delivering intermediate **CXLII**. Subsequent ring closure affords oxonium ion **CXLIII**, which undergoes nucleophilic addition of water at the carbonyl to generate protonated hemiacetal **CXLIV**. A sequence of proton transfer, C─O bond cleavage, and hydrolysis ultimately furnishes product **150**, while regenerating catalyst **C6**.

**SCHEME 50:**
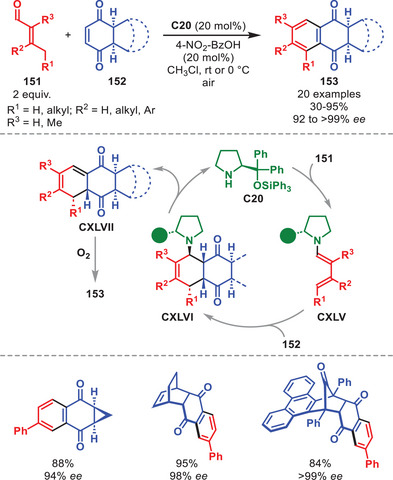
Desymmetrizing oxidative [4+2] cycloaddition between α,β‐unsaturated aldehydes **151** and *meso*‐cyclohexenediones **152**.

In 2022, Mukherjee et al. reported the first application of diarylprolinol silyl ether catalysis to the de novo construction of chiral arenes through a desymmetrizing oxidative [4+2] cycloaddition. The protocol, based on catalyst **C20**, enabled the reaction of α,β‐unsaturated aldehydes **151** with *meso*‐cyclohexenediones **152** which, under aerobic conditions, provided access to enantioenriched, centrally chiral unfunctionalized arenes **153** (Scheme 50) [163]. The transformation proved remarkably general with respect to substitution patterns on both coupling partners. Substituents at the α‐, β‐, or γ‐positions of **151**, as well as cyclic analogous, were all suitable substrates, and *meso*‐quinones **152** fused to rings of different sizes consistently delivered the corresponding **153** with high enantioselectivity. The versatility of the method allowed the synthesis of a range of fused polycyclic architectures, which were shown to be amenable to further synthetic elaboration. The transformation proceeds through a stereoselective, *endo*‐selective [4+2] cycloaddition between dienamine **CXLV** and *meso*‐cyclohexenedione **152**, affording intermediate **CXLVI**, which undergoes facile elimination of catalyst **C20**. The resulting cyclohexadiene **CXLVII** is then converted, via aerobic and stereoablative oxidation, into the enantioenriched arene **153**.

Notably, the same group later extended this strategic concept by employing alkoxy‐directed dienamine catalysis with the same catalyst **C20**, enabling the stereoselective synthesis of benzo‐[3]‐ladderanol [164].

**SCHEME 51:**
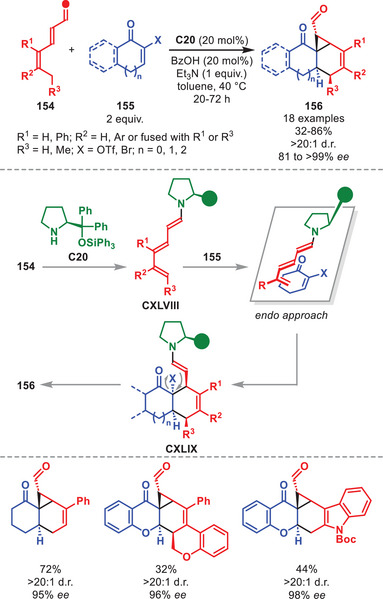
[4+2] Cyloaddition/S_N_2 cascade between dienals **154** and α‐(*pseudo*‐)halogenated enones **155**.

A notable example exploiting trienamine catalysis for the construction of chiral polycyclic architectures was reported in 2021 [165]. This work introduced the strategic integration of the halogen or *pseudo‐*halogen effect—that is, the presence of a halogen or *pseudo*‐halogen substituent in one of the cycloaddition partners to enhance *endo*‐selectivity in the cycloaddition—to promote a diarylprolinol silyl ether–catalyzed Diels–Alder reaction. This approach enabled the stereoselective synthesis of norcarenes **156** (Scheme 51). A panel of dienals **154** and electron‐poor α‐(*pseudo*‐)halogenated enones **155** were successfully engaged in the presence of catalyst **C20**. DFT calculations unveiled the mechanism of the transformation, which is initiated by condensation of **C20** with **154** to generate trienamine **CXLVIII**, which undergoes a step‐wise and highly *endo*‐selective cycloaddition with **155** to afford enamine intermediate **CXLIX**. This species is involved in a cascade sequence by an intramolecular nucleophilic substitution at the leaving group (OTf, or Br), thereby forging the cyclopropane ring embedded in the final norcarene adduct **156**. Using this protocol, a range of norcarene derivatives were isolated in generally high yields and excellent enantioselectivity, invariably as single diastereoisomers. Notably, modulation of the ring size of the *pseudo*‐halogenated enone, possible benzofusion, and the use of diverse acyclic and cyclic trienals—including heteroatom‐tethered variants—enabled access to a collection of previously unexplored, enantioenriched polycyclic scaffolds.

**SCHEME 52:**
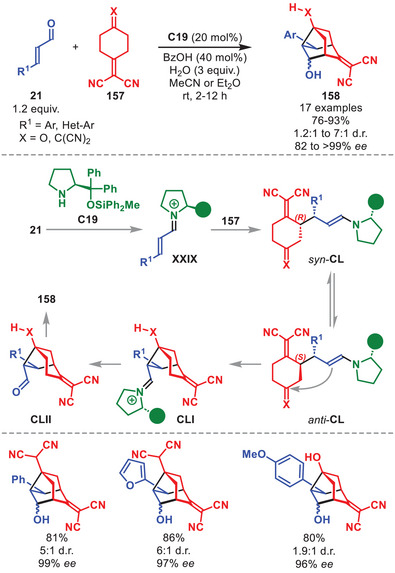
Michael/epimerization/Michael (or aldol)/1,2‐addition between α,β‐unsaturated aldehydes **21** and alkylidene malononitriles **157**.

Turning to LUMO‐lowering iminium‐ion catalysis applied to the enantioselective synthesis of complex polycyclic architectures, a notable example was recently reported by Hayashi et al. In this study, the authors disclosed a diarylprolinol silyl ether–catalyzed domino reaction between α,β‐unsaturated aldehydes **21** and 2,2‐(cyclohexane‐1,4‐diylidene)dimalononitrile or 2‐(4‐oxocyclohexylidene)malononitrile pronucleophiles **157**, catalyzed by **C19** (Scheme 52) [166]. This transformation represented the first enantioselective entry to noradamantane‐based scaffolds **158**, which were obtained with excellent enantioselectivity and moderate to good diastereocontrol (Scheme 52). The protocol proved broadly applicable to a variety of substituted **21**. The domino reaction is initiated by a Michael addition of **157** to iminium ion **XXIX**, in situ generated from **21** and **C19**, furnishing *syn*‐**CL**. Due to the strong electron‐withdrawing character of the methylene malononitrile moiety, this intermediate undergoes facile epimerization, establishing an equilibrium between *syn*‐**CL** and *anti*‐**CL**. The *anti*‐diastereoisomer is the only one geometrically allowed to undergo a subsequent intramolecular cyclization—either a Michael addition (when X = C(CN)_2_) or an aldol reaction (when X = O)—to generate iminium ion **CLI**. Hydrolysis of this intermediate provides aldehyde **CLII**, whose conformation enables a final intramolecular addition to the aldehyde carbonyl group, ultimately forging the enantioenriched noradamantane core embedded in **158**.

**SCHEME 53:**
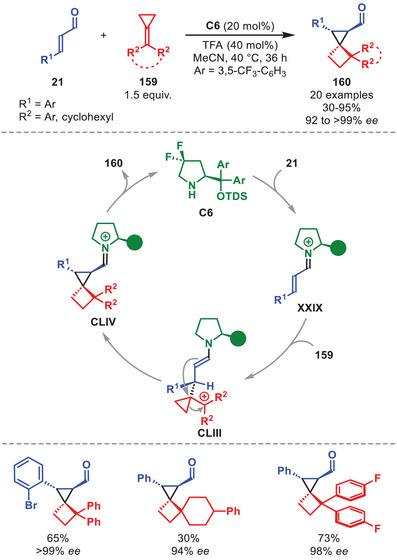
Michael addition/ring expansion/cyclization cascade between α,β‐unsaturated aldehydes **21** and methylenecyclopropanes **159**.

Diarylprolinol silyl ether catalysis has also recently found application in the construction of highly strained spirocyclic architectures. In 2020, Xu et al. reported a general organocatalytic enantioselective strategy for the synthesis of spiro[2,3]hexane frameworks **160** from α,β‐unsaturated aldehydes **21** and methylenecyclopropanes **159** (Scheme 53) [167]. A key element of this approach was the use of an electron‐deficient, *gem*‐difluoro‐substituted catalyst **C6**, which condenses with **21** to generate iminium ion **XXIX**. Subsequently, Michael addition of **159** to the β‐position of iminium ion **XXIX** affords cationic enamine intermediate **CLIII**. This species undergoes an enamine‐mediated ring‐expansion rearrangement, in which opening of the cyclopropane ring leads to formation of a four‐membered ring, while concomitantly generating a new cyclopropane moiety within iminium ion **CLIV**. Hydrolysis of this intermediate regenerates catalyst **C6** and delivers the enantioenriched spiro[2,3]hexane product **160**.

DFT calculations showed that the electrophilic addition of enamine to cyclopropyl cation and rearrangement to the four‐membered ring within **CLIII** occur in a concerted fashion. Moreover, NMR characterization of iminium ions derived from different diarylprolinol silyl ether catalysts revealed that the iminium ion formed from **C6** was more electrophilic, accounting for the reactivity observed. Regarding substrate scope, a range of α,β‐unsaturated aldehydes **21** with various electronic and steric properties, as well as diaryl‐substituted methylenecyclopropanes **159**, were well tolerated. Notably, the methodology could also be extended to access a novel chiral dispiro[2.1.5^5^.1^3^]undecane motif in high enantioselectivity, albeit in low yield.

Some further diarylprolinol silyl ether‐catalyzed rearrangements have been recently reported by the group of Christmann [168] and Vicario [169].

## 8 Conclusion and Outlooks

A distinctive feature of diarylprolinol silyl ethers is the leading role they have played in shaping the evolution of asymmetric aminocatalysis over the past two decades. In this review, we have presented recent advances to highlight new directions in the use of this catalytic platform within the landscape of modern asymmetric organocatalysis.

We have described how classic aminocatalytic activation modes have been combined with photochemistry, thereby unveiling novel activation mode patterns, where the generation of diarylprolinol silyl ether‐tethered intermediates allow for the challenging stereochemical control over open‐shell one‐electron reactivity. Furthermore, recent applications in electrochemistry have been summarized, providing insight into an emerging field that we expect will flourish in the future. The long‐standing synergy with metal catalysis is continuously expanding. Selected examples have been provided, in which various metal‐activated intermediates facilitate novel reactions merged with classic organocatalytic intermediates. We have shown representative examples of enantioselective higher‐order cycloadditions enabled using diarylprolinol silyl ether catalysts to access enantioenriched, complex polycyclic molecular architectures in few synthetic steps. We further described how the maturity of this catalytic platform has manifested in its use within key steps of total synthesis of natural products. Finally, relevant examples employing classical organocatalytic intermediates in novel cycloaddition reactions through unprecedented mechanisms, enabling enantioselective access to new cyclic chemotypes, have been illustrated.

Overall, these reports exemplify the privileged role that diarylprolinol silyl ethers have played over the last decade in disclosing new concepts that have expanded the scope of organocatalysis beyond classical activation modes. We expect further advances in the use of this catalyst class, particularly through the expansion of classical concepts enabled by their integration with emerging technologies and by the creation of increasing molecular complexity in a stereoselective fashion: *A new chapter starts now*.

## Conflicts of Interest

The authors declare no conflicts of interest.

## Data Availability

Data sharing is not applicable to this article as no new data were created or analyzed in this study.
